# Synthesis, Structure, and Reactivity of Magnesium Pentalenides

**DOI:** 10.1021/acs.inorgchem.3c02087

**Published:** 2023-09-15

**Authors:** Hugh J. Sanderson, Gabriele Kociok-Köhn, Ulrich Hintermair

**Affiliations:** † Department of Chemistry, University of Bath, Claverton Down, Bath BA2 7AY, U.K.; ‡ Material and Chemical Characterisation Facility, University of Bath, Claverton Down, Bath BA2 7AY, U.K.; § Institute for Sustainability, University of Bath, Claverton Down, Bath BA2 7AY, U.K.

## Abstract

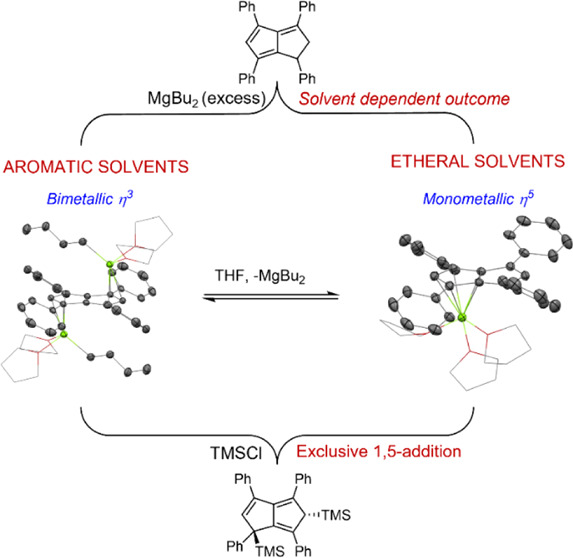

The first magnesium pentalenide complexes have been synthesized *via* deprotonative metalation of 1,3,4,6-tetraphenyldihydropentalene (**Ph**_**4**_**PnH**_**2**_) with magnesium alkyls. Both the nature of the metalating agent and the reaction solvent influenced the structure of the resulting complexes, and an equilibrium between **Mg[Ph**_**4**_**Pn]** and **[**^**n**^**BuMg]**_**2**_**[Ph**_**4**_**Pn]** was found to exist and investigated by NMR, XRD, and UV–vis spectroscopic techniques. Studies on the reactivity of **Mg[Ph**_**4**_**Pn]** with water, methyl iodide, and trimethylsilylchloride revealed that the **[Ph**_**4**_**Pn]**^**2**–^ unit undergoes electrophilic addition at 1,5-positions instead of 1,4-positions known for the unsubstituted pentalenide, **Pn**^**2**–^, highlighting the electronic influence of the four aryl substituents on the pentalenide core. The ratio of *syn*/*anti* addition was found to be dependent on the size of the incoming electrophile, with methylation yielding a 60:40 mixture, while silylation yielded exclusively the *anti*-isomer.

## Abstract 2

The first magnesium pentalenide is reported and shown to exist in two different forms depending on the solvent used. Their interconversion as well reactivity with different electrophiles are reported, showing 1,5-addition on to the tetraphenyl-pentalenide with no influence of the cation on stereoselectivity of the stepwise addition.

## 1 Introduction

Pentalenide (C_8_H_6_^2–^, **Pn**^**2**–^), an aromatic 10π dianion, is the bicyclic analogue of the 6π cyclopentadienyl anion (C_5_H_5_^–^, **Cp**^–^) and related to the monocyclic 10π dianion cyclooctatetraenide (C_8_H_8_^2–^, **COT**^**2**–^)^1^ via a transannular ring closure.^2^ Whereas **Cp**^–^ is ubiquitous in organometallic chemistry, with complexes known for nearly every metal in the periodic table,^3^ and **COT**^**2**–^ chemistry is well-developed for lanthanides and actinides,^4^ pentalenide chemistry is underexplored in comparison. This is often attributed to the difficulty in synthesizing suitable synthons, with the neutral pentalene (**Pn**) being antiaromatic and the nonaromatic dihydropentalene (**PnH**_**2**_) often prone to dimerization or polymerization.^5^ Consequently, pentalenide chemistry has largely been confined to one of three frameworks over the last 60 years—unsubstituted **Pn**^**2**–^,^6^ permethylated [Pn^*^]^**2**–^,^7^ and bis-silylated **[Pn**^**†**^**]**^**2**–^.^8^ Much like **Cp**^–^ chemistry, the synthesis of metal pentalenide complexes often relies on transmetalating salt metathesis of a pentalenide source with a suitable precursor in which the formation of an alkali-metal halide byproduct provides additional driving force for the transmetalation. These are typically salts of lithium (**Li**_**2**_**Pn**, **Li**_**2**_Pn^*^)^6,7^ or potassium (**K**_**2**_**Pn**^**†**^),^8^ although stannylated derivatives have also been used in cases where the group 1 salts were found to be too reducing.^5,9,10^ A result of the relative success of group 1 **Pn**^**2**–^ salts is that the group 2 chemistry of **Pn**^**2**–^ has yet to be explored, again in contrast to **Cp**^–^ and **COT**^**2**–^. A barium dibenzopentalenide has previously been reported;^11^ however, the annulated benzene groups impart greater stability onto the pentalene core, thus obscuring most intriguing properties of pentalene^12^ such as the folding of the ligand seen with d^0^ pentalenide complexes.^13^

The metallocenes of all of the alkaline-earth metals are known,^3^ and magnesocene (**MgCp**_**2**_) has been employed as an alternative to **NaCp** in the synthesis of d- and f-block metallocene complexes.^14−16^**MgCp**_**2**_ exhibits Schlenk equilibria with MgX_2_ to form the corresponding Grignard reagent **CpMgX**.^17^ These Grignards can transfer a single **Cp**^–^ group onto a metal or allow for functionalization of the Cp ring by treatment with electrophiles.^18^ Most of the group 2 **COT**^**2**–^ chemistry has focused on the heavier alkaline-earth metals,^4^ with triple decker sandwich complexes of the type **[(Cp’Ae)**_**2**_**COT]** (Cp’ = ^i^Pr_4_Cp; Ae = Ca, Sr, Ba)^19,20^ and the inverse amido sandwich complexes **[{(Me**_**3**_**Si)**_**2**_**NAe(THF)***_**x**_***}**_**2**_**COT]** (Ae = Ca (*x* = 1), Sr (*x* = 2))^21^ reported. **MgCOT** has also been prepared and employed in transmetalations^22,23^ as well as reactions with chlorophosphines where after initial electrophilic addition, the (bisphosphino)cycloocta-1,3,5-triene undergoes either a ring opening to yield 1,8-(bisphosphino)octa-1,3,5,7-tetraene or isomerization to 7,8-bisphopshino-bicyclo[4.2.0]octa-2,4-diene depending on the R groups on the chlorophosphine.^24^**MgCOT** has also been shown to react with dichlorophosphines to form phosphindole derivatives and other organophosphorous compounds.^25^ The structure of **MgCOT** is still unknown, and its identity has only been inferred from the analysis of hydrolysis products or other qualitative assessments, however.^26^ Given its small size and lack of accessible d orbitals, Mg^2+^ is unlikely to bind η^8^ to **COT**^**2**–^ like the heavier alkaline earths do.^4^ Indeed, in the XRD structure of **[(**^**Dipp**^**NacNac)Mg]**_**2**_**COT** (Dipp = 2,6-^i^Pr_2_C_6_H_3_) Mg^2+^ is bound η^2^ to **COT**^**2**–^.^27^ Thus, with regard to pentalenide chemistry, the alkaline earths in general and magnesium in particular present two intriguing questions: first, the nature of bonding between the d^0^ metal and **Pn**^**2**–^, and second, how might the divalent cation influence *syn* or *anti* selectivity in transmetalations compared to the commonly used *anti*-**A**_**2**_**Pn** salts (A = Li or K).

Recently, we have reported the synthesis of a tetra-arylated dihydropentalene (**1,3,4,6-Ph**_**4**_**PnH**_**2**_) and its deprotonative metalation with a range of group 1 bases to afford the first examples of an arylated pentalenide including the first sodium pentalenide complex.^28^ The homobimetallic salts were found to be of low solubility, with higher solubility achieved through the formation of heterobimetallic salts such as **Li·K[Ph**_**4**_**Pn]**. However, over time, the heterobimetallic salts were found to undergo cation migration in solution and precipitate the less soluble homobimetallic salts. Given these challenges posed by the group 1 **[Ph**_**4**_**Pn]**^**2**–^ salts and the success of prior group 2 **Cp**^–^ and **COT**^**2**–^ chemistry, attention was turned to the hitherto unexplored alkaline-earth chemistry of **[Ph**_**4**_**Pn]**^**2**–^. Herein, we describe the synthesis of the first magnesium pentalenide complex, its solvent dependence on formation, and its reactivity toward electrophiles.

## 2 Results and Discussion

### 2.1 Syntheses and Structures of Magnesium Pentalenides

**Figure 1:**
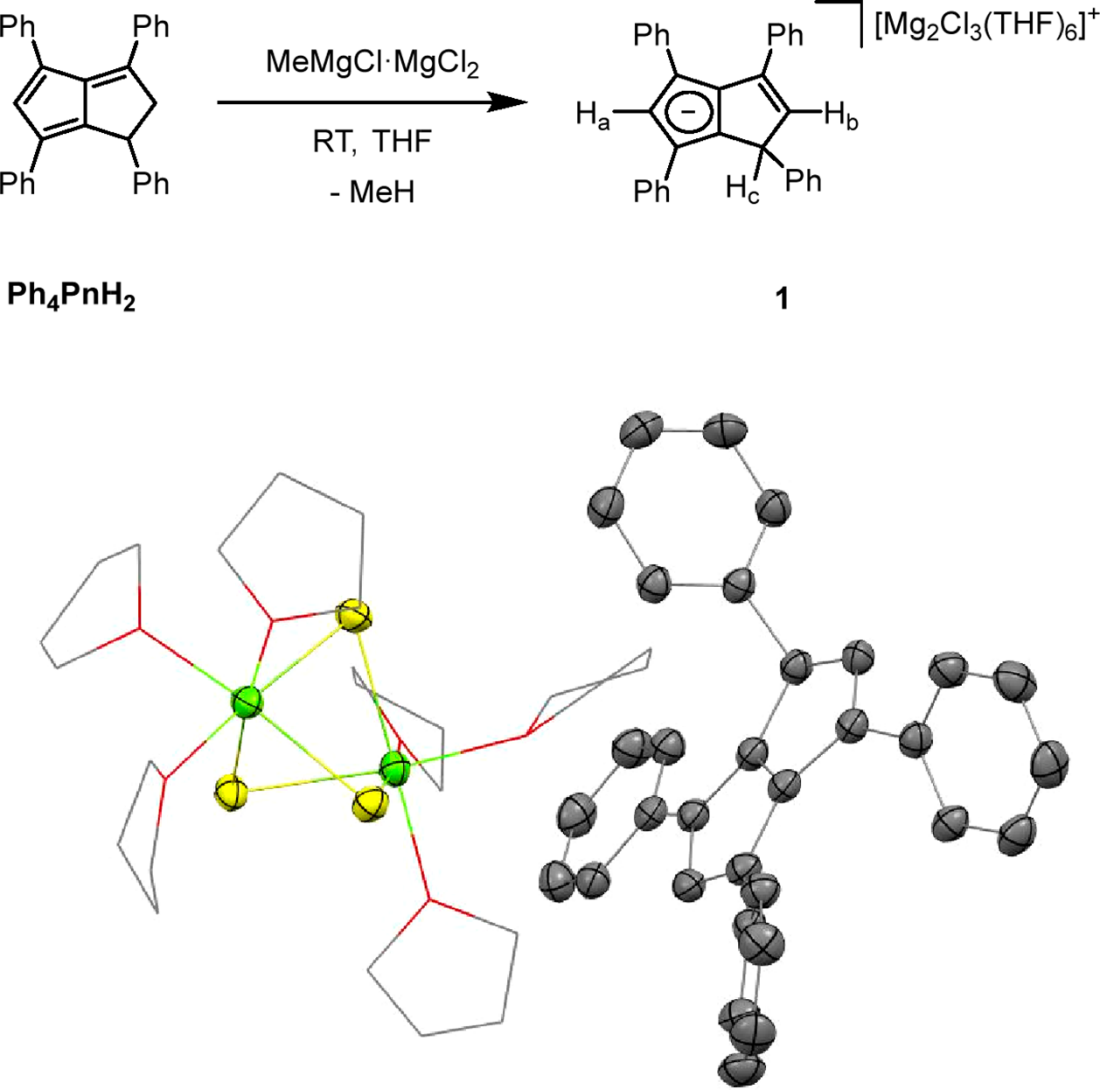
Synthesis of [Mg_2_(*μ*-Cl)_3_(THF)_6_][Ph_4_PnH] (**1**) (top) and its X-ray crystal structure with thermal ellipsoids at the 50% probability level (bottom; hydrogen atoms omitted for clarity).

Previous studies on the stepwise deprotonation of **Ph**_**4**_**PnH**_**2**_ revealed its first pK_A_ of ∼15 to access the formation of **[Ph**_**4**_**PnH]**^–^, which exhibits a second p*K*_A_ of ∼25 to afford **[Ph**_**4**_**Pn]**^**2**–^.^28^ Therefore, commercially available alkyl magnesium complexes having a p*K*_A_ ≥30 (dependent on the nature of the alkyl substituent) can serve as suitable starting points for magnesium pentalenide chemistry. Indeed, the addition of MeMgCl to a THF solution of **Ph**_**4**_**PnH**_**2**_ resulted in the gradual consumption of dihydropentalene and formation of a hydropentalenide (**1**) within 24 h (Figure 1, top). The ^1^H NMR spectrum of **1** contained three characteristic signals at 6.50, 5.79, and 4.59 ppm, corresponding to H_a_, H_b_, and H_c_, with associated ^13^C signals at 105.2, 131.9, and 52.7 ppm. These observations were very similar to previously reported values for the group 1 (Li/Na/K) **[Ph**_**4**_**PnH]**^–^ salts in THF,^28^ suggesting that **1** exists as a solvent-separated ion pair (SSIP) in solution.

Crystals suitable for XRD were grown by the addition of hexane to a THF solution of **1**. In agreement with the NMR observations, the solid-state structure of **1** revealed a solvent-separated ion pair with two magnesium atoms bridged by three chloride atoms and further solvated by three THF molecules each. The observation of [Mg_2_(μ-Cl)_3_(THF)_6_]^+^ instead of the stoichiometrically expected [MgCl(THF)_5_]^+^ in the XRD structure of **1** is attributed to the presence of excess MgCl_2_ in commercial Grignard solutions. This cationic cluster has been reported before and is of interest in the field of magnesium batteries.^29^ However, in ethereal solutions of **1**, there likely exists a dynamic mixture of a variety of cationic magnesium chloride species. The C–C distances of the anionic 6π ring in the noncontact **[Ph**_**4**_**PnH]**^–^ ranged between 1.403(2) and 1.430(3) Å, while the C–C distances in the nonaromatic ring varied between 1.351(3) and 1.522(3) Å, as expected for localized C=C and C–C bonds and consistent with those found in **K[Ph**_**4**_**PnH]**.^28^ In this case, the preference of magnesium to bind to hard donors resulted in an ion pair instead of a direct magnesium pentalenide interaction.

When an excess amount (5–20 equiv) of MeMgCl was used, **Ph**_**4**_**PnH**_**2**_ first underwent full conversion to **[Ph**_**4**_**PnH]**^–^ within 24 h, and over the course of 3 weeks further conversion of **[Ph**_**4**_**PnH]**^–^ was noticed with the emergence of a single signal for both wingtip protons (**H**_**w**_) at 6.80 ppm and four equivalent phenyl groups in the aromatic region indicative of **[Ph**_**4**_**Pn]**^**2**–^. To accelerate the reaction and avoid the presence of hard halide donors to bind to magnesium, the related dialkylmagnesium reagent ^n^Bu_2_Mg was used in place of MeMgCl. Pleasingly, stirring a dark red THF solution of **Ph**_**4**_**PnH**_**2**_ with a stoichiometric amount or slight excess of ^n^Bu_2_Mg at room temperature gave a bright red solution over the course of 24 h that slowly precipitated an orange solid after 4–5 days. The ^1^H NMR spectrum of the solution saw the disappearance of characteristic signals corresponding to **Ph**_**4**_**PnH**_**2**_ and the appearance of a singlet assigned to **H**_**w**_ at 6.80 ppm along with four equivalent phenyl groups again indicative of a *D*_2*h*_ symmetrical **[Ph**_**4**_**Pn]**^**2**–^. As in the case of **1**, the resemblance of the spectrum to that of the previously reported heterobimetallic **Li·K[Ph**_**4**_**Pn]** suggests that in solution, **Mg[Ph**_**4**_**Pn]** (**2**) exists as a solvent-separated ion pair, which was further supported by DOSY experiments (Figure S9). The orange precipitate could be redissolved in THF and was also found to be pure **2** by NMR spectroscopy. Following the reaction by ^1^H NMR indicated that the formation of **2** proceeded through a hydropentalenide intermediate with peaks characteristic of **[Ph**_**4**_**PnH]**^–^ seen at 4.75, 5.96, and 6.52 ppm (Figure S7). The use of TMEDA, either instead of THF or as a cosolvent, resulted in significantly shorter reaction times (less than 2 h when used neat) presumably due to kinetic activation of ^n^Bu_2_Mg.^30^ The observation that the analogous reaction of **Ph**_**4**_**PnH**_**2**_ with ^n^BuLi led to decomposition^28^ may be attributed to the relative hardness of Li^+^*versus* Mg^2+^. Complex **2** was found to be moderately soluble in coordinating solvents such as THF, pyridine, DME, and TMEDA, sparingly soluble in aromatics such as benzene and toluene, and practically insoluble in hydrocarbons such as hexane and pentane.

**Figure 2:**
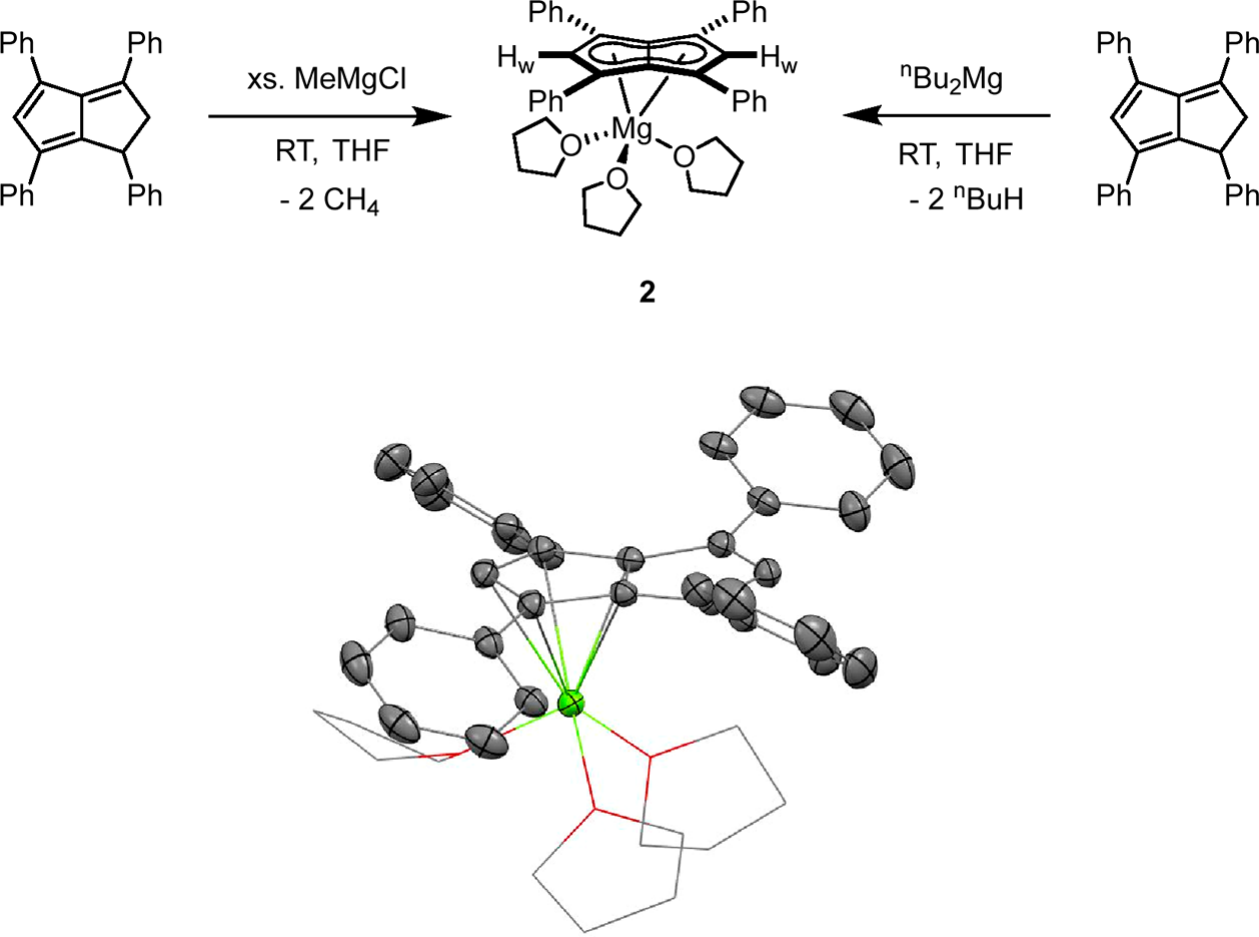
Synthesis of [Mg(THF)_3_][Ph_4_Pn] (**2**) (top) and its X-ray crystal structure with thermal ellipsoids at the 50% probability level (bottom; hydrogen atoms omitted for clarity).

Standing a THF solution of **2** at −35 °C yielded orange crystals suitable for XRD analysis. The solid-state structure of **2** showed the magnesium cation to sit preferentially over one C_5_ ring in an η^5^ manner (Figure 2). The shortest magnesium-centroid (C_t_) distance found (2.0839[15] Å) was 5% longer than the Mg-C_t_ and Li-C_t_ distances in **MgCp**_**2**_ (1.98[1] Å)^31^ and **Li**_**2**_**[Ph**_**4**_**Pn]** (1.9785[1] Å),^28^ and the wingtip carbon-C_5_-centroid-metal (C_w_-C_t_-M) angle of 94.1° was more obtuse than in **Li**_**2**_**[Ph**_**4**_**Pn]** where the cations sat centrally over each C_5_ ring at exactly 90.0°. The parameter Δ can be used to quantify the extent of deviation from η^5^ to η^3^ as a measure of ring slippage for **Cp**^–^ and indenyl complexes, and is defined as the difference in the average M-C_B_ and M-C_NB_ distances (C_NB_ = non-bridgehead carbons).^32−34^ Applying this analysis to **2** returns a value of Δ = −0.07, which further supports the slight deviation from perfect η^5^ toward the bridgehead carbons, likely a reflection of the divalent magnesium feeling an electrostatic attraction from both C_5_ rings. The cation was further solvated by three THF molecules in a *pseudo*-tetrahedral geometry with an average C_t_-Mg–O angle of 123.3° and O–Mg–O angle of 92.7°. The apparent discrepancy of the symmetry of **2** in the solid state and in solution is due to its dissociation into a SSIP when dissolved in a donor solvent such as THF, and no changes in the ^1^H NMR spectra could be observed down to −60 °C (Figure S8) as previously found for the heterobimetallic group 1 salts of **[Ph**_**4**_**Pn]**^**2**–^.^28^

**Figure 3:**
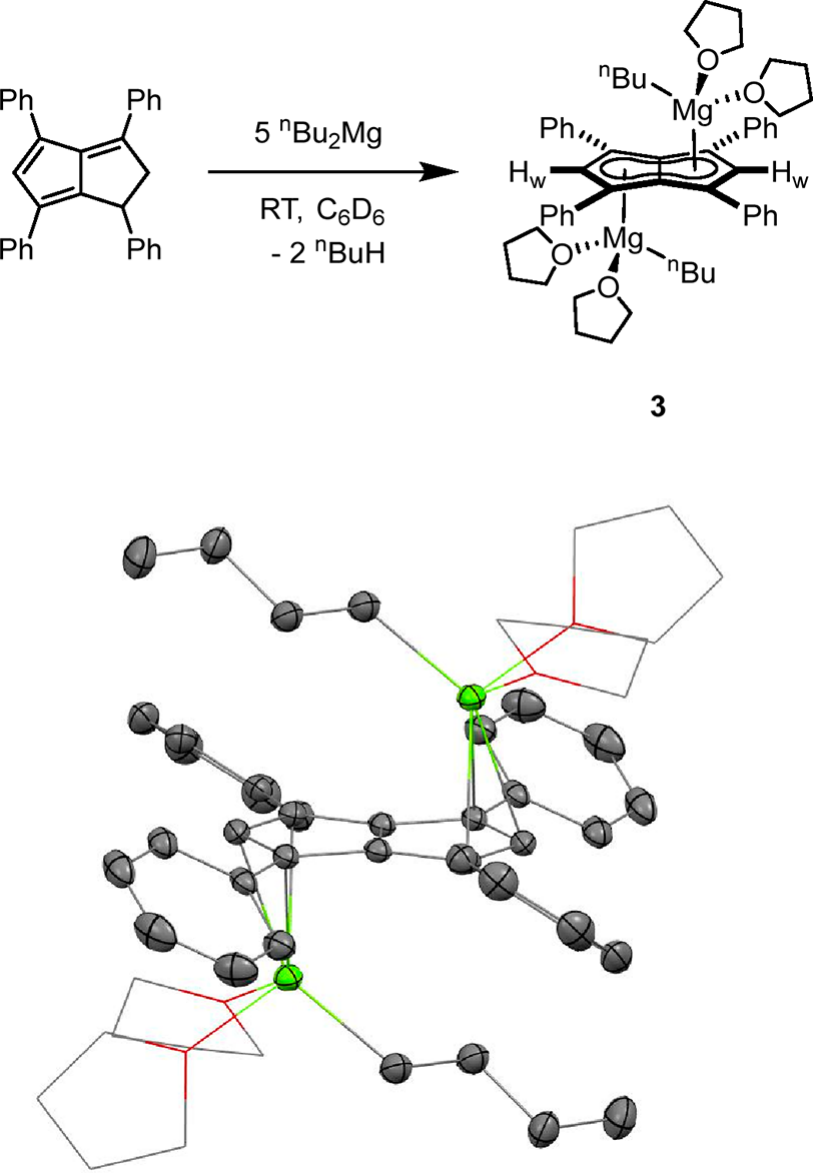
Synthesis of [Mg^n^Bu(THF)_2_]_2_[Ph_4_Pn] (**3**) (top) and its X-ray crystal structure with thermal ellipsoids at the 50% probability level (bottom; hydrogen atoms omitted for clarity).

In an attempt to synthesize a donor-free magnesium pentalenide complex, the reaction medium was changed from ethereal to aromatic solvents. Addition of ^n^Bu_2_Mg to a benzene solution of **Ph**_**4**_**PnH**_**2**_ led to a color change from dark red to dark yellow over the course of a week at room temperature. The *in situ*^1^H NMR spectrum of the reaction mixture revealed complicated signals partially attributable to dihydropentalene-type systems (Figure S13). Addition of hexane to this solution led to the formation of bright orange crystals, which upon redissolution in benzene showed a symmetrical **[Ph**_**4**_**Pn]**^**2**–^ environment with **H**_**w**_ shifted to be overlapping with the meta protons of the phenyl groups in the range of 7.34–7.31 ppm. Signals assigned to a ^n^butyl group could also be identified via a broad α-CH_2_ peak at −0.20 ppm. XRD analysis of these crystals showed an *anti* bimetallic magnesium pentalenide **[Mg**^**n**^**Bu(THF)**_**2**_**]**_**2**_**[Ph**_**4**_**Pn]** (**3**) where the bound THF must have originated from the commercial ^n^Bu_2_Mg reagent (Figures S14 and 15). The solid-state structure shown in Figure 3 revealed a planar **[Ph**_**4**_**Pn]**^**2**–^ system coordinated to two equivalent ^n^BuMg(THF)_2_ units, indicating that only one alkyl group of each ^n^Bu_2_Mg had reacted with the dihydropentalene. The Mg^2+^ cations in **3** were bound to **[Ph**_**4**_**Pn]**^**2**–^ in a more η^3^ coordination than in **2**, as evidenced by a Mg-C_t_-C_w_ angle of 81.7, 12° more acute than in **2** and increased Mg-C_B_ bond lengths 0.32 Å longer in **3** than in **2**. The Mg^2+^ also sat 0.2 Å further away from the C_5_ rings in **3**, as evidenced by the increased Mg-C_t_ distance of 2.2525(7) Å. **3** displayed a ring slippage value of Δ = 0.23, which is slightly less than that reported for η^3^ bis(indenyl)magnesium (Δ = 0.27).^35,36^ The geometry around each Mg^2+^ center in **3** is still best described as pseudo-tetrahedral, with marginally more acute O–Mg–O angles (86.5°) in comparison to **2** (89.6–95.3°).

The observed difference in reactivity of ^n^Bu_2_Mg toward **Ph**_**4**_**PnH**_**2**_ in THF and benzene can be attributed to different levels of aggregation of the alkyl magnesium complex in these solvents.^30^ In comparison to **2**, **3** was found to be soluble in aromatic solvents such as benzene and toluene to give yellow solutions (Figure S20). THF solutions of **2** were orange in color and displayed a strong UV–vis absorbance at 354 nm (ε = 60,800 M^–1^ cm^–1^) and a less intense band at 300 nm (ε = 32,830 M^–1^ cm^–1^). A weak, broad absorption around 480 nm was also seen, responsible for the orange/red color of these solutions as reported for **Li·K[Ph**_**4**_**Pn]**.^28^ In contrast, the yellow solutions of **3** in C_6_D_6_ exhibited weak bands at 307 nm (ε = 4320 M^–1^ cm^–1^) and 357 nm (ε = 9,360 M^–1^ cm^–1^) only. When solid **3** was dissolved in a donor solvent such as THF, however, orange solutions with more intense UV–vis signatures at 312 nm (ε = 47,050 M^–1^ cm^–1^), 383 nm (ε = 34,520 M^–1^ cm^–1^), 428 nm (ε = 36,240 M^–1^ cm^–1^), and 520 nm (ε = 78,429 M^–1^ cm^–1^) were obtained (Figure S16). Removing the solvent *in vacuo* yielded an orange solid, and crystallization of the orange compound formed from dissolving yellow **3** in THF showed it to be the mono-Mg complex **2** by XRD (Figure S42).

**Scheme 1:**
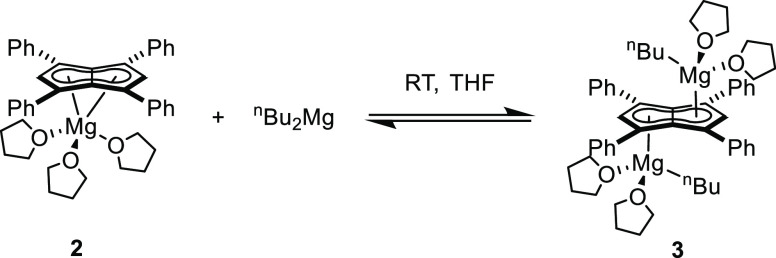
Interconversion of [Mg(THF)_3_][Ph_4_Pn] (**2**) and [MgBu(THF)_2_]_2_[Ph_4_Pn] (**3**)

Like many organomagnesium compounds, **Mg(Cp)**_**2**_ is known to engage in dynamic ligand exchange with dialkylmagnesiums (MgR_2_) in donor solvents to form mixed **CpMgR** complexes.^37,38^ An analogous equilibrium is thus likely responsible for the observed formation of **2** by dissolving **3** in THF (Scheme 1). To probe this, a sample of isolated **2** was treated with excess ^n^Bu_2_Mg in THF and the UV–vis spectrum recorded. After addition of ^n^Bu_2_Mg, the main absorption around 360 nm disappeared and two new bands at 406 and 480 nm formed, indicating a shift in the solution speciation toward **[MgBu]**_**2**_**[Ph**_**4**_**Pn]** (Figure S17). In addition to the symmetrical **[Ph**_**4**_**Pn]**^**2**–^ system, the NMR spectra of these solutions showed characteristic peaks of 1-butene (Figure S21), indicative of β-hydride elimination and magnesium hydride formation during (or in parallel to) the interconversion of **2** and **3**.^39^ Finally, when **3** was dissolved in a large excess of THF, the ^1^H NMR showed the characteristic signals of **2** alongside the presence of ^n^Bu_2_Mg (Figure S18).

### 2.2 Reactivity Toward Electrophiles

**Scheme 2:**
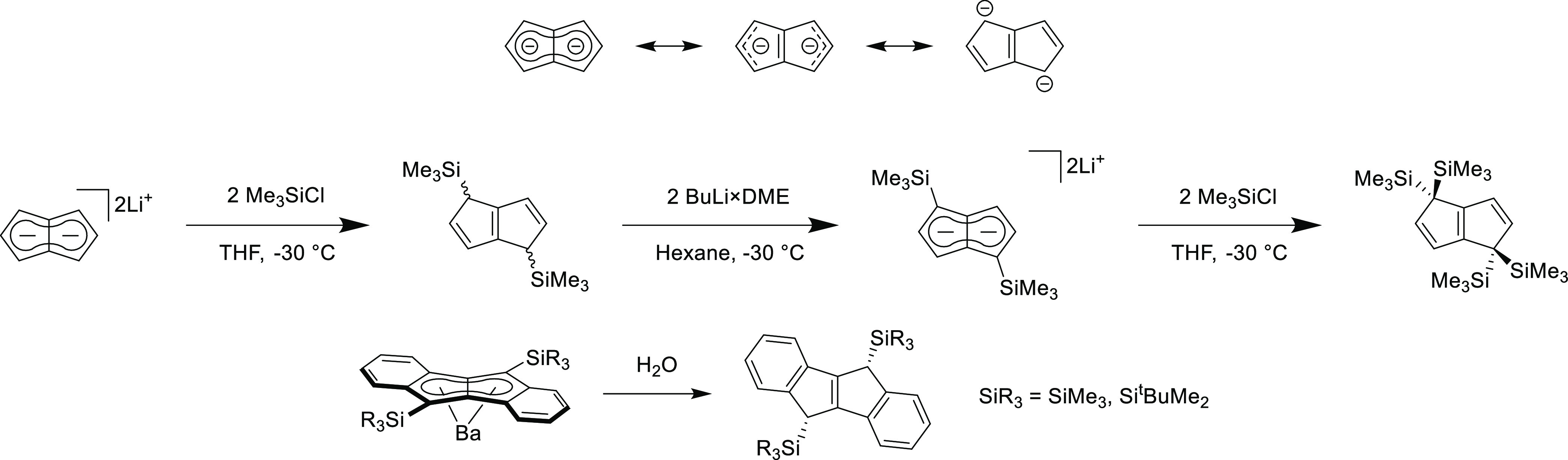
Charge Resonance Forms of Unsubstituted Pentalenide (Top),^13,40^ Synthesis of 1,4-Bis(TMS)pentalenide (Middle),^8^ and Synthesis of 5,10-Bis(trialkylsilyl)-5,10-dihydro-dibenzopentalene (Bottom)^11^

**Pn**^**2**–^ is known to have a resonance form where the negative charges are located at 1,4-carbons,^13,40^ which results in the formation of η^1^-substituted complexes at the C1/C4-positions (Scheme 2 top).^9,10,41−43^ For example, trialkylsilyl groups add to unsubstituted **Pn**^**2**–^ at the 1,4-positions as a mixture of *syn* and *anti* products, which may undergo further deprotonation and electrophilic addition of another two TMS groups again at 1,4-positions.^8^ Xi and co-workers observed that the hydrolysis of disilylated barium dibenzopentalenide yielded *syn*-dibenzodihydropentalene, posited to be due to the Ba^2+^ cation blocking one face of the anion.^11^

In this context, to understand the behavior of electrophiles toward **Mg[Ph**_**4**_**Pn]**, the reactivity of complex **2** with chosen electrophiles H_2_O, D_2_O, MeI, and TMSCl was investigated. The reactions were complete within minutes in THF at room temperature in all cases, and key NMR assignments of the products obtained are summarized in Table 1. Hydrolysis of **2** gave quantitative conversion to the corresponding 1,5-dihydropentalene, as evident by signals at 6.49 and 4.93 ppm assigned to H_a_ and H_b_, respectively, and the distinctive coupling of the geminal CH_2_ group at the 5-position. In comparison to **2** (H_w_ = 6.80 ppm, C_b_ = 109.5 ppm), the observed shifts in the signal corresponding to H_a_ and C_b_ (50.8 ppm) clearly indicated the change in hybridization from sp^2^ to sp^3^. The identity of the hydrolysis product was further confirmed by using D_2_O, which showed 95% D-incorporation in the 5 (b)-position. The signal assigned to H_a_ remained consistent at 6.49 ppm, demonstrating that H_a_ was H_w_ from **2**. No ^2^H NMR shifts or H–D coupling constants could be resolved, however, and the peak of ^13^C_c_ was too weak to be observed due to ^2^H–^13^C coupling.

**Table 1:**
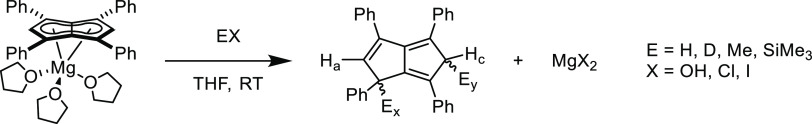

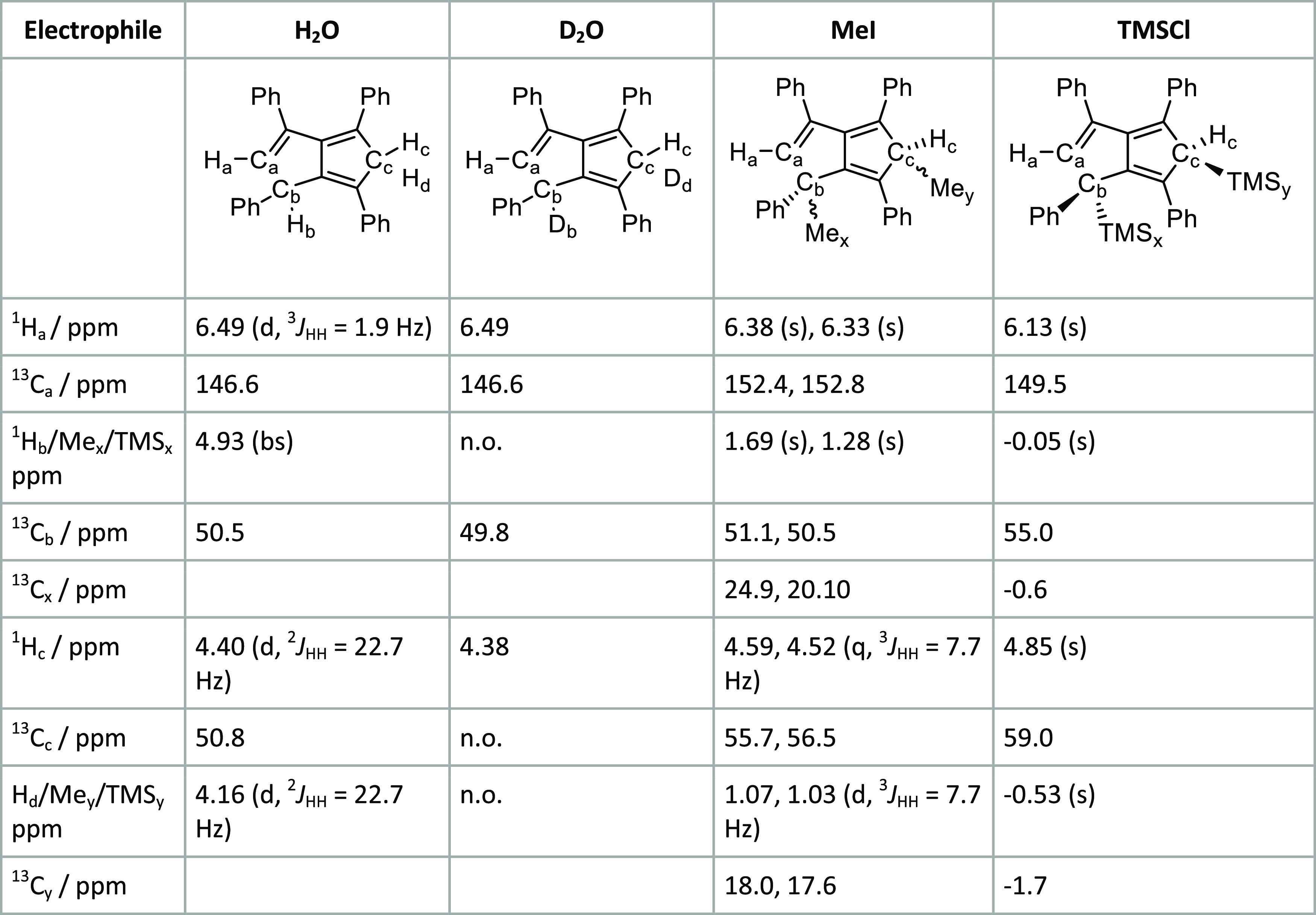
Key NMR Assignments from the Electrophilic Attack on **Mg[Ph**_**4**_**Pn]** (**2**) by Water, MeI, and TMSCl (n.o. = Not Observed)

In order to probe whether a 1,4-dihydropentalene had perhaps formed initially and then rapidly isomerized to the observed 1,5-isomer, electrophiles irreversibly forming strong σ bonds were employed. Using larger substituents with diagnostic NMR signatures also allowed for probing the stereochemistry of the addition reaction. Addition of an excess of methyl iodide to **2** in THF led to an immediate color change from orange to pale yellow alongside precipitation of MgI_2_. The organic product of the reaction showed a pair of ^1^H singlets at 6.39 and 6.33 ppm, with associated ^13^C signals at 152.4 and 152.7 ppm, respectively, which were assigned to H_a_ based on the shifts noted for 1,3,4,6-Ph_4_-1,5-PnH_2_ from the hydrolysis of **2**. Two quartets at 4.59 and 4.52 ppm were assigned as H_c_, coupling with a methyl group, and the associated ^13^C shifts at 55.7 and 56.5 ppm were indicative of a sp^3^ carbon environment. The geminal H–C–CH_3_ arrangement was further supported by 2D NMR spectroscopy (Figure S33). The fact that two signals were also found for each of the two methyl groups Me*_x_* and Me*_y_* (see Table 1), but mass spectrometry confirmed the formation of a dimethylated product, confirmed the formation of a racemic mixture of both *syn*- and *anti*-diastereomers of a 1,5-addition product in an approximate 60:40 (or 40:60) ratio. This slight deviation from an equimolar ratio suggested that the electrophilic addition proceeded in a stepwise manner *via* a hydropentalenide-type intermediate, posing the question as to whether attack occurred first at the 1- or 5-position. However, attempts to probe this by using one equivalent of MeI resulted in the consumption of half an equivalent of **2** and formation of the same 1,5 di-addition product, possibly due to the enthalpic driving force of MgX_2_ formation. The same observations were made with the larger electrophile TMS, except that in this case only one diastereoisomer formed. No cross-peaks between the two TMS groups in the 1- and 5-positions were observable in NOESY experiments, strongly suggesting the exclusive formation of the *anti*-isomer due to the increased steric bulk of the TMS group compared to CH_3_, leading to steric repulsion during the stepwise addition. This observation is consistent with what O’Hare and co-workers reported for the formation of *anti*-**1,4-(Me**_**3**_**Sn)**_**2**_Pn*.^10^ However, while they were able to generate the corresponding *syn*-stannylated isomer *via* kinetic trapping of a proposed *syn*-**Li**_**2**_Pn* intermediate using nonpolar solvents,^10^ in our case, the same *anti*-**1,5-(Me**_**3**_**Si)**_**2**_**[Ph**_**4**_**Pn]** was formed when the reaction of **2** with TMSCl was carried out in toluene. Furthermore, when we used *anti*-**Li**_**2**_**[Ph**_**4**_**Pn]** in place of **2**, the same product was found again (Figure S41), showing that in these SSIPs, the countercation(s) had no impact on the stereoselectivity of the substitution. The observed *syn*-1,4-diprotonation of Xi’s barium pentalenide is likely due to the different electronics of the annulated silylbenzopentalenide compared to **[Ph**_**4**_**Pn]**^**2**–^ and/or a reflection of the higher covalency and relative softness of Ba^2+^ over Mg^2+^.

## 3 Conclusions

We have described the isolation of the first magnesium pentalenide complex **Mg[Ph**_**4**_**Pn]** from the straightforward deprotonative metalation of **Ph**_**4**_**PnH**_**2**_ with a commercially available dialkylmagnesium. The same complex could also be obtained by the reaction of **Ph**_**4**_**PnH**_**2**_ with an excess of Grignard reagents but with concomitant formation of magnesium-halide clusters. Compared to the homobimetallic group 1 **[Ph**_**4**_**Pn]**^**2**–^ salts (Li/Na/K), **Mg[Ph**_**4**_**Pn]** showed improved solubility in coordinating solvents such as THF and pyridine, and unlike the metastable heterobimetallic group 1 salts, **Mg[Ph**_**4**_**Pn]** can also be isolated, stored, and redissolved without change. These properties make it a convenient starting point to explore the p-, d-, and f-block chemistry of arylated pentalenides. A solvent dependence on the formation of η^5^**Mg[Ph**_**4**_**Pn]** was found, with the use of aromatic solvents in place of THF, resulting in the formation of the η^3^ dimagnesium complex *anti*-**[**^**n**^**BuMg]**_**2**_**[Ph**_**4**_**Pn]**. In ethereal solvents, both complexes were found to interconvert in a manner typical of other organomagnesium reagents.^37,38^ Despite their structural differences, complexes **Mg[Ph**_**4**_**Pn]**, *anti*-**[**^**n**^**BuMg]**_**2**_**[Ph**_**4**_**Pn]**, and *anti*-**Li**_**2**_**[Ph**_**4**_**Pn]** all reacted analogously upon exposure to electrophiles to afford the 1,5-addition product instead of the 1,4-addition reported for *anti*-**Li**_**2**_**[Pn]**.^8^ This result shows that **[Ph**_**4**_**Pn]**^**2**–^ reacts independently of the nature of the cation, consistent with its solvent-separated ion pair structure in solution. The formation of a 1,5-addition product suggests that in **[Ph**_**4**_**Pn]**^**2**–^, charge localization is highest at the 1- and 5-positions, suggesting that the nature of the substituents on **Pn**^**2**–^ heavily influences the electronics of the system.⊥ The steric bulk of the incoming electrophile appears to be the largest factor that determines *syn*/*anti* selectivity, with small electrophiles (H_2_O, D_2_O, MeI) giving approximately equimolar mixtures of *syn* and *anti*-isomers, whereas the bulkier TMS group exclusively yielded the *anti*-isomer. These insights will be useful for the targeted synthesis of new pentalenide complexes with controlled stereochemistry.

## 4 Experimental Section

### 4.1 General Considerations

All reactions were conducted under argon using standard Schlenk techniques or a MBraun Unilab Plus glovebox unless stated otherwise. All commercially available materials were purchased from Sigma-Aldrich, Fisher, or Acros.

### 4.2 Solvents

Methanol was dried and distilled over magnesium. Toluene was dried and distilled over sodium. THF, hexane, and pentane were dried and distilled over potassium. C_6_D_6_ was distilled over CaH_2_ and stored over 4 Å molecular sieves. TMEDA, DME, and 1,4-dioxane were distilled over CaH_2_.

### 4.3 Reagents

1,3,4,6-Tetraphenyl-1,2-dihydropentalene (Ph_4_PnH_2_) and dilithium 1,3,4,6-tetraphenylpentalenide (Li_2_[Ph_4_Pn]) were prepared according to literature procedures.^28^ TMSCl and MeI were freshly distilled over CaH_2_ prior to use.

### 4.4 Analysis

NMR spectra were obtained using a 500 MHz Bruker Avance III at 25 °C unless stated otherwise. Chemical shifts (δ) are given in ppm and referenced to residual proton chemical shifts from the NMR solvent for ^1^H and ^13^C{^1^H} spectra. UV–vis spectroscopy was performed inside a MBraun Unilab Plus glovebox using a fiber-optic AvaSpec-2048L photospectrometer with an AvaLight-DH-S-BAL light source and 400 μm cables (Avantes). Data was collected between 250 and 1000 nm with an integration time of 4 ms.

Single-crystal X-ray diffraction analysis was carried out using a RIGAKU SuperNova, Dual,Cu a zero EoS2 single-crystal diffractometer. Mass spectrometry was carried out at the Material and Chemical Characterization Facility at the University of Bath using a Bruker MaXis HD ESI-QTOF.

#### 4.4.1 Synthesis of [Mg_2_(μ-Cl)_3_(THF)_6_][Ph_4_PnH] (**1**)

Ph_4_PnH_2_ (0.100 g, 0.24 mmol) was dissolved in 1 mL of THF and to this, MeMgCl (0.160 mL of a 2.53 M THF solution, 0.40 mmol) was added. The reaction was left stirring at room temperature for 36 h. Next, the solution was concentrated to 2 mL and washed with *n*-hexane (3 × 10 mL) to afford the product as a red powder, which was dried under vacuum (0.086 g, 32%).
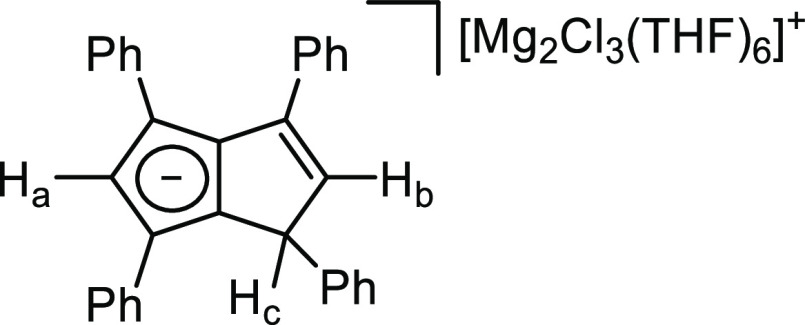
^**1**^**H NMR** (500 MHz, DMSO-D_6_) δ: 7.27–7.23 (m, 7H, ArH), 7.12–7.09 (m, 6H, ArH), 7.00 (t, ^3^*J*_HH_ = 7.1 Hz, 2H, ArH), 6.92 (d, ^3^*J*_HH_ = 7.5 Hz, 2H, ArH), 6.86 (t, ^3^*J*_HH_ = 7.6 Hz, 3H, ArH), 6.80 (t, ^3^*J*_HH_ = 7.5 Hz, 3H, ArH), 6.63 (t, ^3^*J*_HH_ = 7.3 Hz, 1H, ArH), 6.51 (t, ^3^*J*_HH_ = 7.3 Hz, 1H, ArH), 6.29 (s, 1H, H_a_), 5.65 (d, ^3^*J*_HH_ = 1.4 Hz, 1H, H_b_), 4.49 (d, ^3^*J*_HH_ = 1.4 Hz, 1H, H_c_), 3.61–3.58 (m, 30H, THF), 1.77–1.74 (m, 30H, THF).

^**13**^**C{**^**1**^**H} NMR** (126 MHz, DMSO-D_6_) δ: 144.0, 143.9, 142.1, 141.5, 133.6, 129.1 (C_b_), 127.9, 127.8, 127.8, 127.6, 127.4, 127.3, 126.7, 126.26, 125.8, 124.8, 122.4, 119.9, 118.7, 115.9, 115.1, 108.5 (C_a_), 67.0 (THF) 51.5 (C_c_), 25.1 (THF).

^**1**^**H NMR** (500 MHz, THF-H_8_) δ: 7.36 (d, ^3^*J*_HH_ = 7.6 Hz, 2H, ArH), 7.34–7.32 (m, 2H, ArH), 7.26 (d, ^3^*J*_HH_ = 7.5 Hz, 2H, ArH), 7.07–7.01 (m, 6H, ArH), 6.92 (t, ^3^*J*_HH_ = 7.1 Hz, 2H, ArH), 6.87–6.80 (m, 3H, ArH), 6.65 (t, ^3^*J*_HH_ = 7.1 Hz, 1H, ArH), 6.54–6.50 (m, 2H, ArH, H_a_), 5.79 (s, 1H, H_b_), 4.59 (s, 1H, H_c_).

^**13**^**C{**^**1**^**H} NMR** (126 MHz, THF-H_8_) δ: 144.8, 144.6, 142.4, 141.8, 140.9, 134.0, 131.8 (C_b_), 129.9, 129.0, 128.7, 128.4, 128.0, 127.8, 127.5, 127.1, 125.9, 125.2, 124.1, 121.2 120.1, 116.7, 115.4, 105.7* (C_a_), 52.9 (C_c_).

*Found by ^1^H-^13^C HSQC.

**HR APCI-MS (−)***m*/*z* expected for [M−Mg_2_Cl_3_(THF)_6_] = 407.1805, found 407.1749.

#### 4.4.2 Synthesis of [Mg(THF)_3_][Ph_4_Pn] (**2**)

##### 4.4.2.1 Method A

1,3,4,6-Tetraphenyl-1,2-dihydropentalene (0.122 g, 0.3 mmol) was dissolved in THF (2 mL) and to this, dibutylmagnesium (0.5 mL of a 1 M heptane solution, 0.5 mmol) was added. The solution was stirred at room temperature for 48 h, after which the solvent was removed *in vacuo* to give an orange powder that was washed with *n*-hexane (2 × 5 mL) and redissolved in 5 mL of THF. Pentane (15 mL) was added and the resulting mixture was left to stand overnight at −35 °C. The supernatant was filtered off and the orange solid washed again with *n*-hexane (2 × 3 mL). The solid was then dried *in vacuo* for 3 h to afford the product as an orange powder (0.110 g, 57%). Crystals suitable for XRD could be grown from standing of a THF solution at −35 °C.

##### 4.4.2.2 Method B

1,3,4,6-Tetraphenyl-1,2-dihydropentalene (0.020 g, 5 mmol) was dissolved in THF (0.5 mL) and to this, MeMgCl (0.1 mL of a 3 M THF solution, 15 mmol) was added and the reaction monitored by ^1^H NMR for 2 weeks. During this time, a color change from dark opaque red to bright transparent red was observed. Formation of **2** was confirmed by ^1^H and ^13^C{^1^H} NMR. Crystals suitable for XRD could be grown from standing of a THF solution at −35 °C alongside crystals of MgCl_2_(THF)_4_.
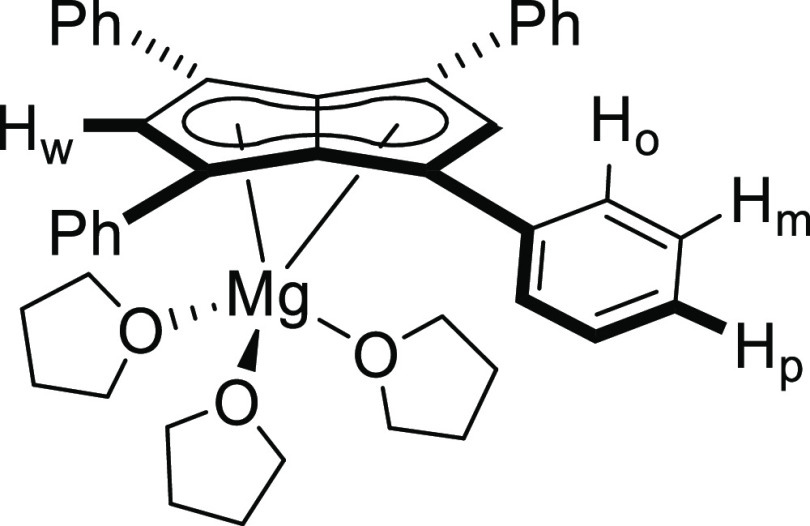
^**1**^**H NMR** (500 MHz, THF-H_8_) δ: 7.13 (d, ^3^*J*_HH_ = 7.5 Hz, 8H, H_o_), 6.95 (t, ^3^*J*_HH_ = 7.4 Hz, 9H, H_m_), 6.80 (s, 2H, H_w_), 6.59 (t, ^3^*J*_HH_ = 7.1 Hz, 4H, H_p_).

^**13**^**C{**^**1**^**H} NMR** (126 MHz, THF-H_8_) δ: 142.1 (C_i_), 127. 2 (C_o_), 126.7 (C_m_), 120.9 (C_B_), 118.8 (C_p_), 115.5 (C_w_), 109.1 (C_q_).

**HR APCI-MS (+)***m*/*z* expected for [M + H] = 647.3370, found 647.4543.

**UV–vis (THF)** λ: 300 nm (ε = 32,830 M^–1^ cm^–1^), 354 nm (ε = 60,800 M^–1^ cm^–1^).

#### 4.4.3 Synthesis of [Mg^n^Bu(THF)_2_]_2_[Ph_4_Pn]

Ph_4_PnH_2_ (0.200 g, 0.49 mmol) was dissolved in 5 mL of C_6_H_6_ and to this, ^n^Bu_2_Mg (2.5 mL of a 1 M heptane solution, 2.45 mmol) was added. After 1 week, hexane (10 mL) was added, and the solution was made to stand at −35 °C overnight and an orange microcrystalline solid formed. The supernatant was removed and the solid dried under vacuum to afford [Mg^n^Bu(THF)_2_]_2_[Ph_4_Pn] as a yellow orange powder (0.089 g, 21% yield). Crystals suitable for XRD could be grown by addition of hexane to a benzene solution and standing at RT.
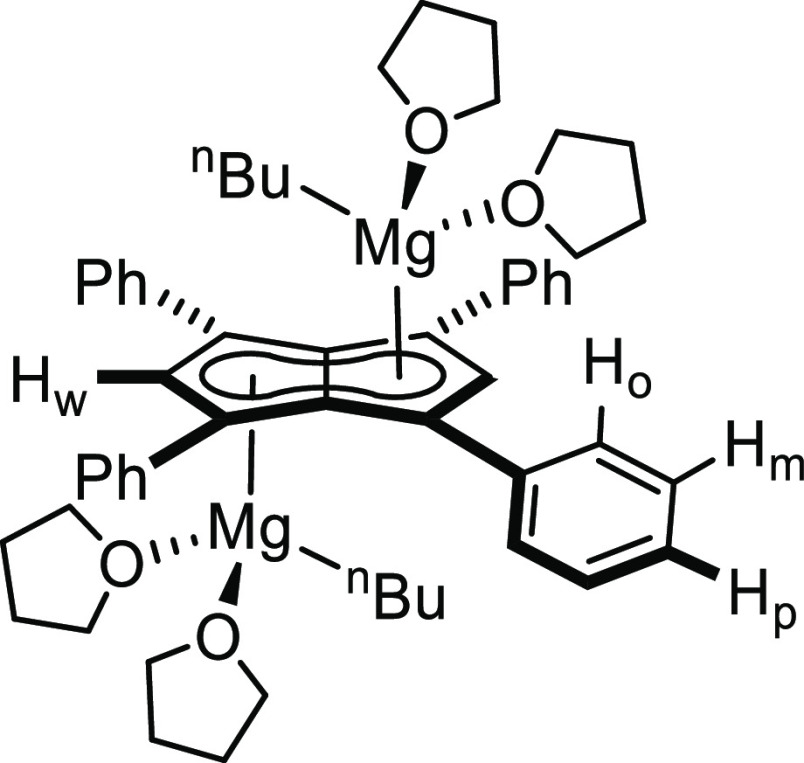


^**1**^**H NMR** (500 MHz, C_6_D_6_) δ: 7.64 (d, ^3^*J*_HH_ = 7.5 Hz, H_o_), 7.34–7.31 (m, H_w_, H_m_), 7.05 (t, ^3^*J*_HH_ = 7.4 Hz, H_p_), 1.81 (bs, ^n^Bu), 1.73–1.69 (m, ^n^Bu), 1.26 (t, ^3^*J*_HH_ = 7.13 Hz, ^n^Bu), −0.20 (bs, ^n^Bu).

^**13**^**C{**^**1**^**H} NMR** (126 MHz, C_6_D_6_) δ: 140.4, (C_i_), 128.3 (C_o_), 127.6 (C_m_), 121.9 (C_p_), 121.6 (C_B_), 115.2 (C_w_), 107.0 (C_q_), 33.5 (^n^Bu), 32.6 (^n^Bu), 14.7 (^n^Bu), 8.2 (^n^Bu).

**UV–vis (THF)** λ = 312 nm (ε = 47,050 M^–1^ cm^–1^), 383 nm (34,520 M^–1^ cm^–1^), 428 nm (36,240 M^–1^ cm^–1^), 520 nm (78,429 M^–1^ cm^–1^).

**UV–vis (C**_**6**_**D**_**6**_**)** λ = 307 nm (ε = 4320 M^–1^ cm^–1^), 357 nm (9360 M^–1^ cm^–1^).

#### 4.4.4 General Procedure for the Reaction with Electrophiles

Mg[Ph_4_Pn] (20 mg, 0.03 mmol) was dissolved in THF (0.5 mL) and to this, 0.1 mL of the electrophile was added. An immediate color change from orange to dark red (for H_2_O and D_2_O) or pale yellow (MeI or TMSCl) was observed along with complete dissolution of Mg[Ph_4_Pn]. In the case of MeI, a white precipitate of MgI_2_ formed within minutes. The products were identified *in situ* using multinuclear 1D and 2D NMR techniques supported by mass spectrometry. Key assignments are given in Table 1 and the original NMR spectra can be found in Figures S22–S41.

Hydrolysis: **HR APCI-MS (−)***m*/*z* expected for [M – H] = 407.1800, found 407.1770.

Deuteration: **HR APCI-MS (−)***m*/*z* expected for [M – H] = 409.1920, found 409.1883.

Silylation: **HR APCI-MS (−)***m*/*z* expected for [M – H] = 551.2596, found 551.3044.
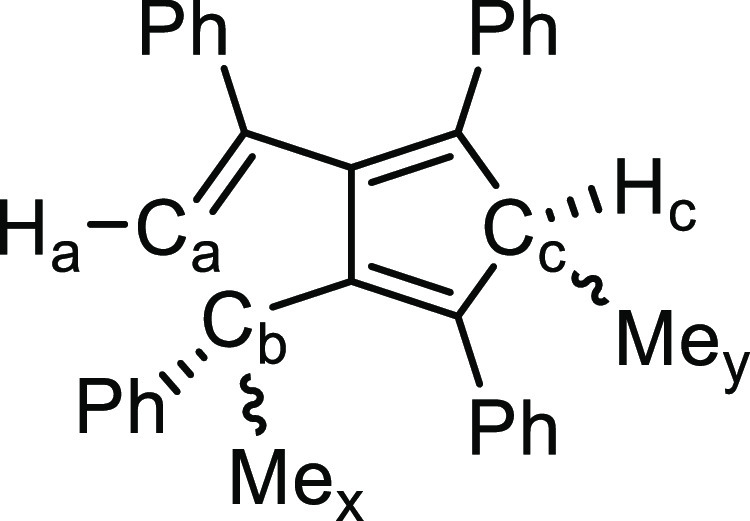


^**1**^**H NMR** (500 MHz, THF-H_8_) δ: 7.51–7.00 (m, 42H, ArH), 6.38 (s, 1H, H_a_), 6.33 (s, 1H, H_a_), 4.59 (q, ^3^*J*_HH_ = 7.75 Hz, 1H, H_c_), 4.52 (q, ^3^*J*_HH_ = 7.75 Hz, 1H, H_c_), 1.69* (Me*_x_*), 1.23* (Me*_x_*) 1.07 (d, ^3^*J*_HH_ = 7.75 Hz, 3H, Me_b_), 1.04 (d, ^3^*J*_HH_ = 7.75 Hz, 2H, Me_b_).

^**13**^**C{^1^H} NMR** (126 MHz, THF-H_8_) δ^†^: 153.9, 153.5, 152.8 (C_a_), 152.4 (C_a_), 146.5, 146.0, 143.6, 140.2, 139.6, 138.6, 138.5, 138.2, 138.2, 136.2, 136.0, 135.9, 135.9, 135.8, 130.3, 130.3, 129.3, 128.9, 128.7, 128.6, 128.5, 128.5, 128.3, 128.1, 128.1, 127.9, 127.8, 127.7, 127.1, 127.0, 126.9, 126.8, 126.7, 126.5, 126.5, 126.5, 56.5 (C_c_), 55.7 (C_c_), 51.1 (C_b_), 50.5 (C_b_), 24.9 (Me*_x_*), 20.1 (Me*_x_*), 18.0 (Me*_y_*), 17.6 (Me*_y_*).

*Found by HSQC.

^†^Due to significant overlap, only 48 signals can be distinguished at this field strength.

**HR APCI-MS (+)***m*/*z* expected for [M + H] = 437.2264, found 437.2252.

## Supporting Information Available

Additional analytical data (NMR, UV–vis, XRD) (PDF)

## Footnotes

⊥ More detailed analyses of the electronic structures of substituted pentalenides will be communicated separately in due course.
