# Supplementary material for: Synthesis, Structure, and Reactivity of Magnesium Pentalenides
Source: Inorg Chem. 2023 Sep 15;62(39):15983–91. doi: 10.1021/acs.inorgchem.3c02087 (PMC10548416; doi:10.1021/acs.inorgchem.3c02087)
Supplement: Supplementary file 1 — ic3c02087_si_001.pdf [file ic3c02087_si_001.pdf]

Supporting Information

Synthesis, Structure and Reactivity of a Magnesium Pentalenide

Hugh J. Sanderson,[a] Gabriele Kociok-Köhn,[b] Ulrich Hintermair*[a,c]

a) Department of Chemistry, University of Bath, Claverton Down, Bath BA2 7AY, UK.
b) Material and Chemical Characterisation Facility, University of Bath, Claverton Down, Bath BA2 7AY, UK.
c) Institute for Sustainability, University of Bath, Claverton Down, Bath, BA2 7AY, UK.
*u.hintermair@bath.ac.uk

Contents

Spectroscopic Data 2
Magnesium 1,3,4,6-tetraphenylhydropentalenide $[Mg_2Cl_3][Ph_4PnH]$ (1) 2
Magnesium 1,3,4,6-tetraphenylpentalenide $[Mg(THF)_3][Ph_4Pn]$ (2) 4
Di(butylmagnesium) 1,3,4,6-tetrapenhylpentalenide $[Mg(^nBu)(THF)_2][Ph_4Pn]$ (3) 9
Commercial Dibutlymagnesium (1M in heptanes) 12
Interconversion Between $[Mg(THF)_3][Ph_4Pn]$ and $[Mg(^nBu)(THF)_2]_2[Ph_4Pn]$ 14
Butene Formation 18
Hydrolysis of Magnesium 1,3,4,6-tetraphenylpentalenide 19
Deuteration of Magnesium 1,3,4,6-tetraphenylpentalenide 24
1,5-dimethyl-1,3,4,6-tetraphenyl-5-hydropentalene 27
1,5-di(trimethyl)silyl-1,3,4,6-tetraphenyl-5-hydropentalene 32
Crystallographic Data 36
Magnesium 1,3,4,6-tetraphenylhydropentalenide $[Mg_2Cl_3][Ph_4PnH]$ (1) 36
Magnesium 1,3,4,6-tetraphenylpentalenide $[Mg(THF)_3]$[Ph4Pn] (2) 37
Di(butylmagnesium) 1,3,4,6-tetraphenylpentalenide $[Mg(^nBu)(THF)_2]_2[Ph_4Pn]$ (3) 38
Magnesium 1,3,4,6-tetraphenylpentalenide from interconversion 39

Spectroscopic Data

Magnesium 1,3,4,6-tetraphenylhydropentalenide $[Mg_2Cl_3(THF)_6][Ph_4PnH]$ (1)

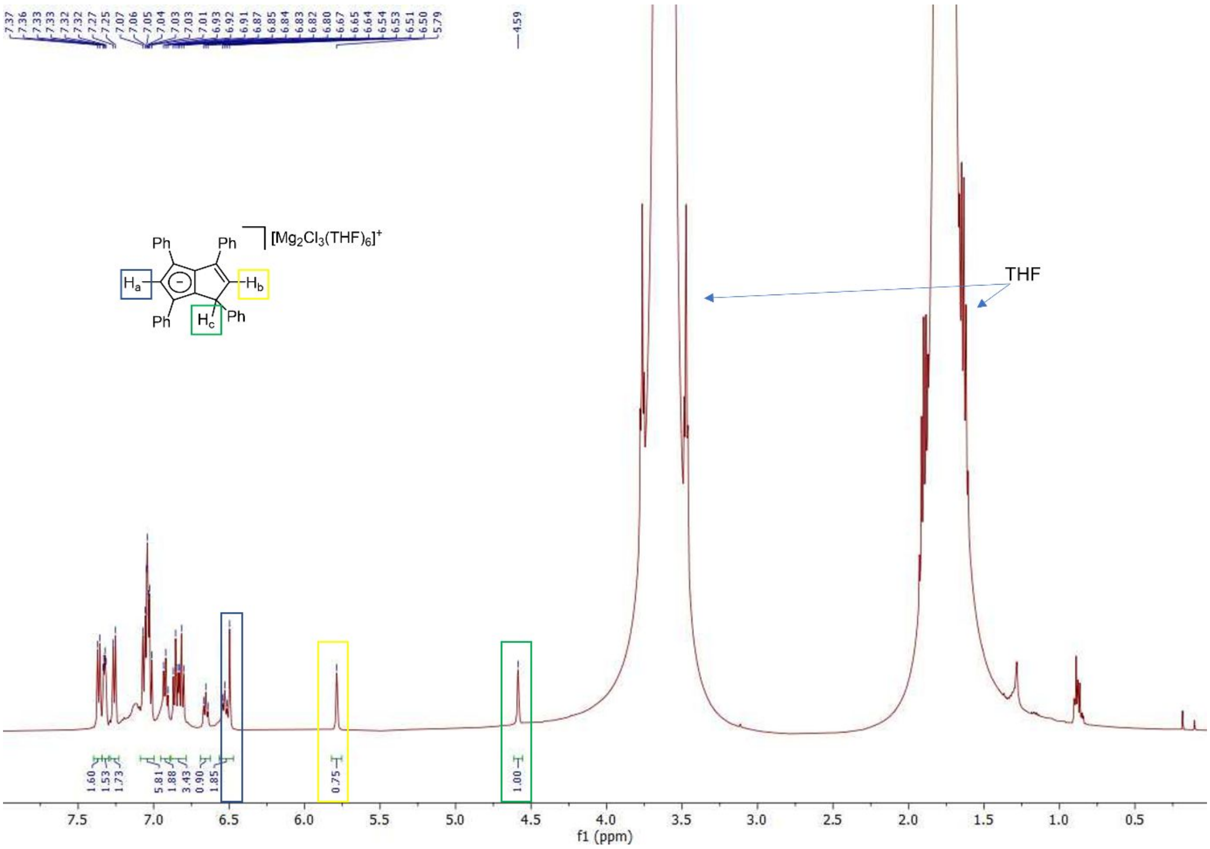

Figure S1: 500 MHz ^1H NMR of $[Mg_2Cl_3(THF)_6][Ph_4PnH]$ in THF-H_8

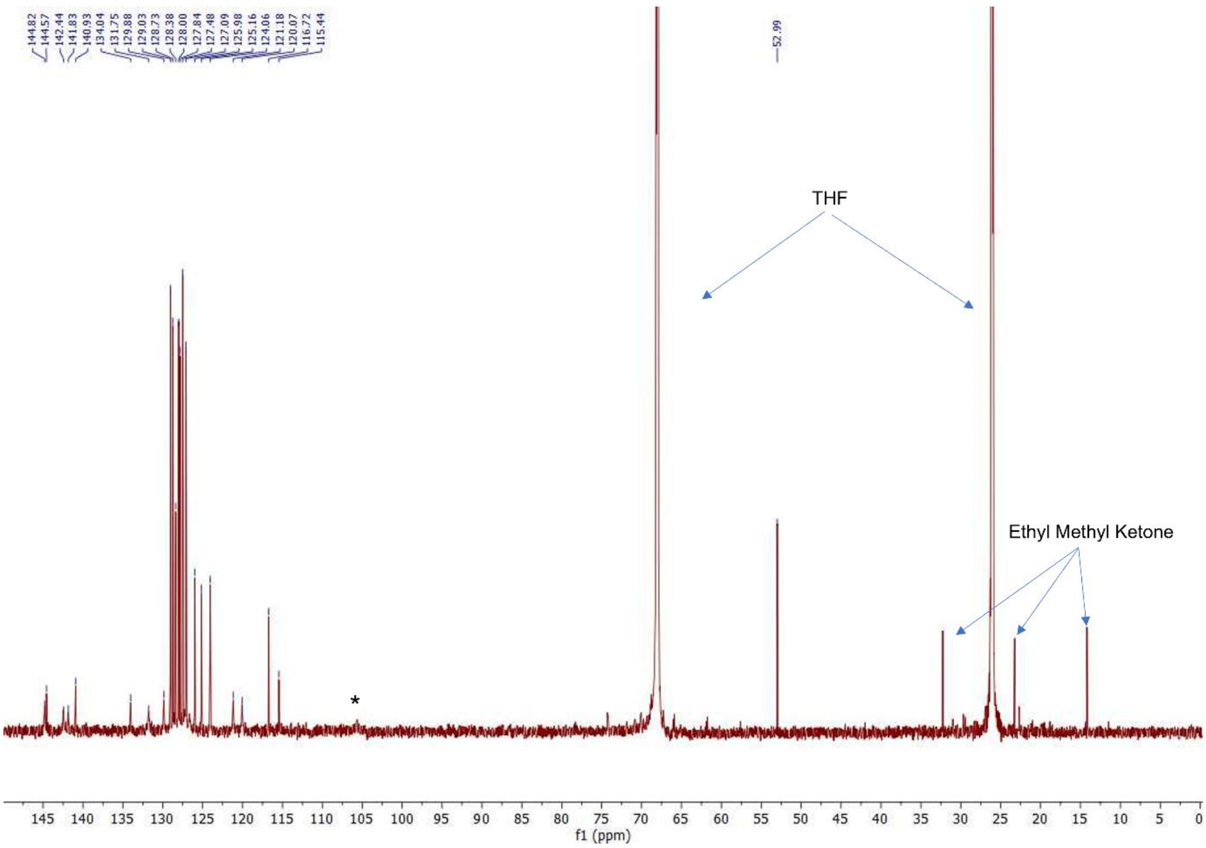

Figure S2: 126 MHz $^{13}C\{^{1}H\}$ NMR of $[Mg_2Cl_3(THF)_6][Ph_4PnH]$ in THF-H_8. *Signal identified by ^{1}H-^{13}C HSQC.

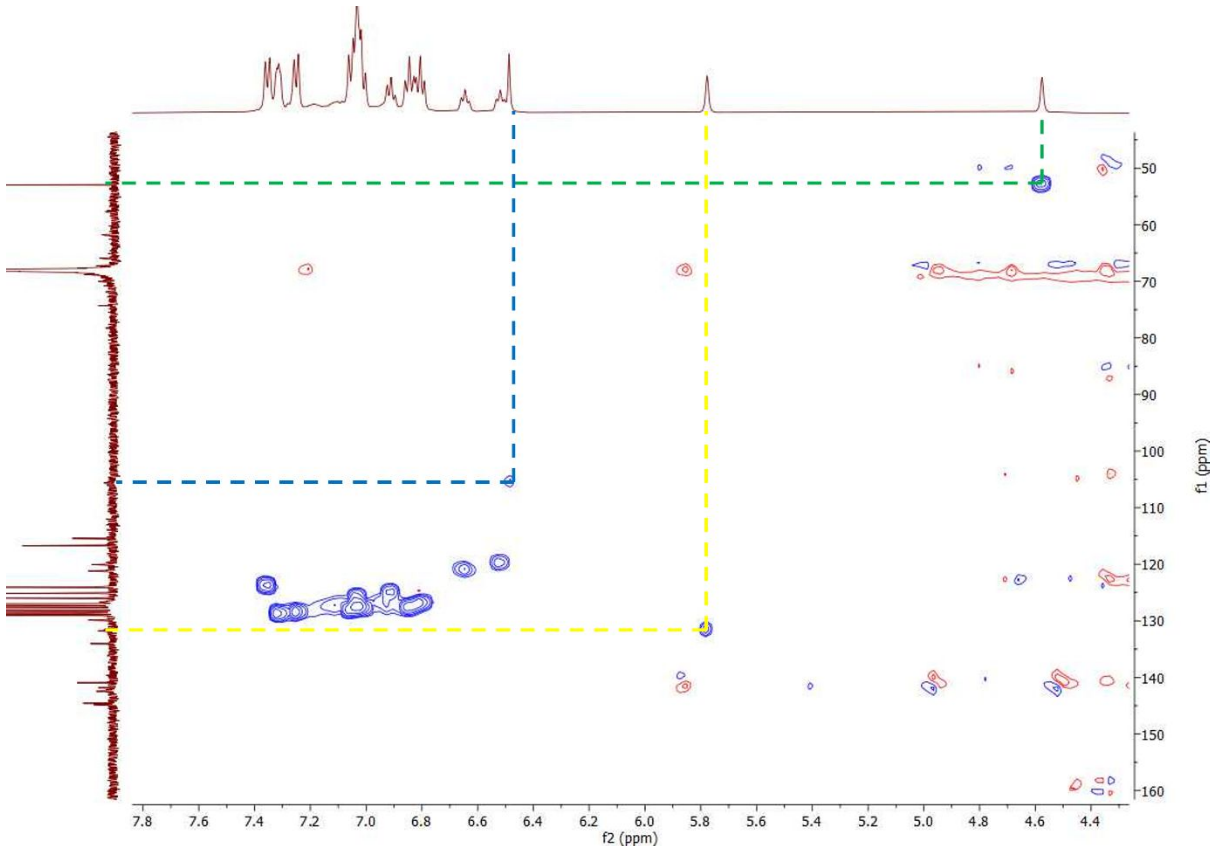

Figure S3: 500 MHz ^{1}H-^{13}C HSQC spectrum of $[Mg_2Cl_3(THF)_6][Ph_4PnH]$ in THF-H_8 (Blue = H_a, Yellow = H_b, Green = H_c)

Magnesium 1,3,4,6-tetraphenylpentalenide [Mg(THF)$_3$][Ph$_4$Pn] (2)

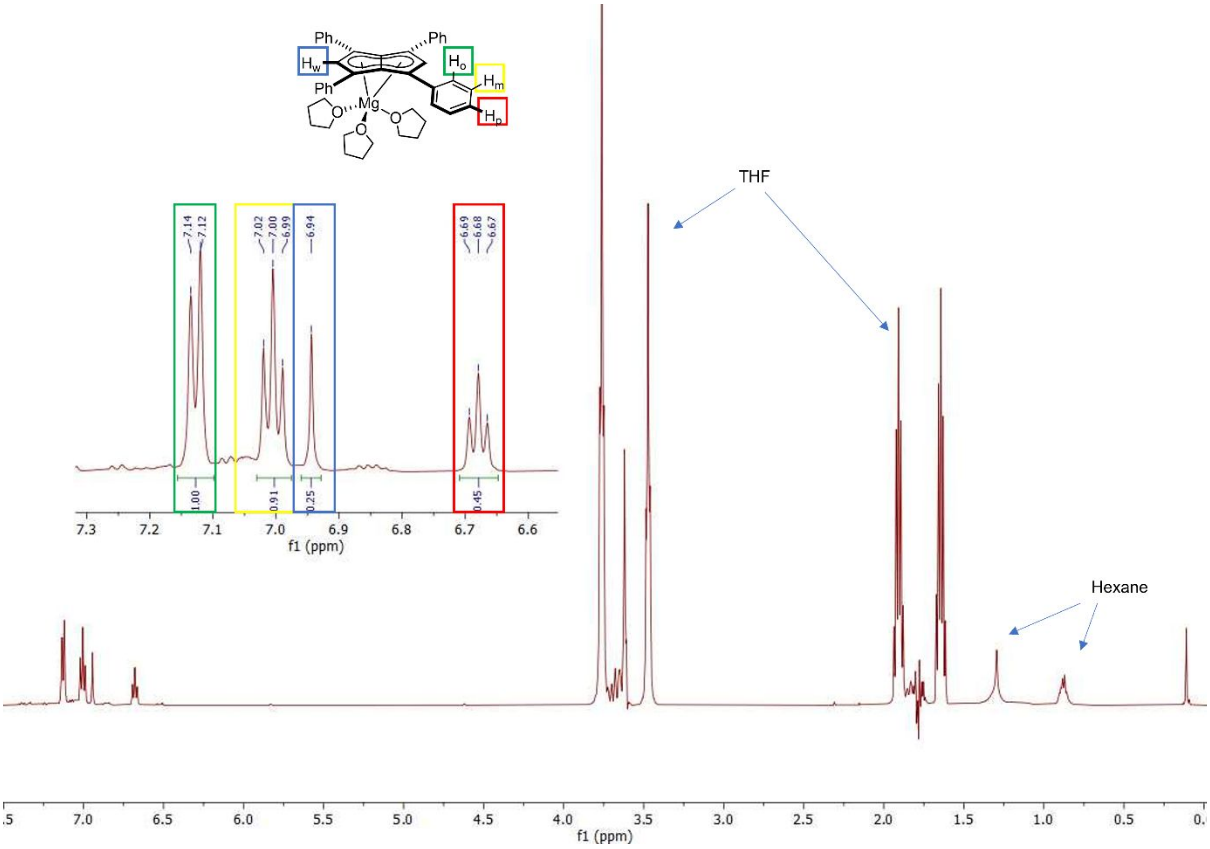

Figure S4: 500 MHz ^{1}H NMR of [Mg(THF)$_3$][Ph$_4$Pn] in THF-H$_8$. Spectrum obtained using the lc1gppnf2 solvent suppression pulse sequence, a double presaturation experiment during relaxation and mixing time using two independent channels.

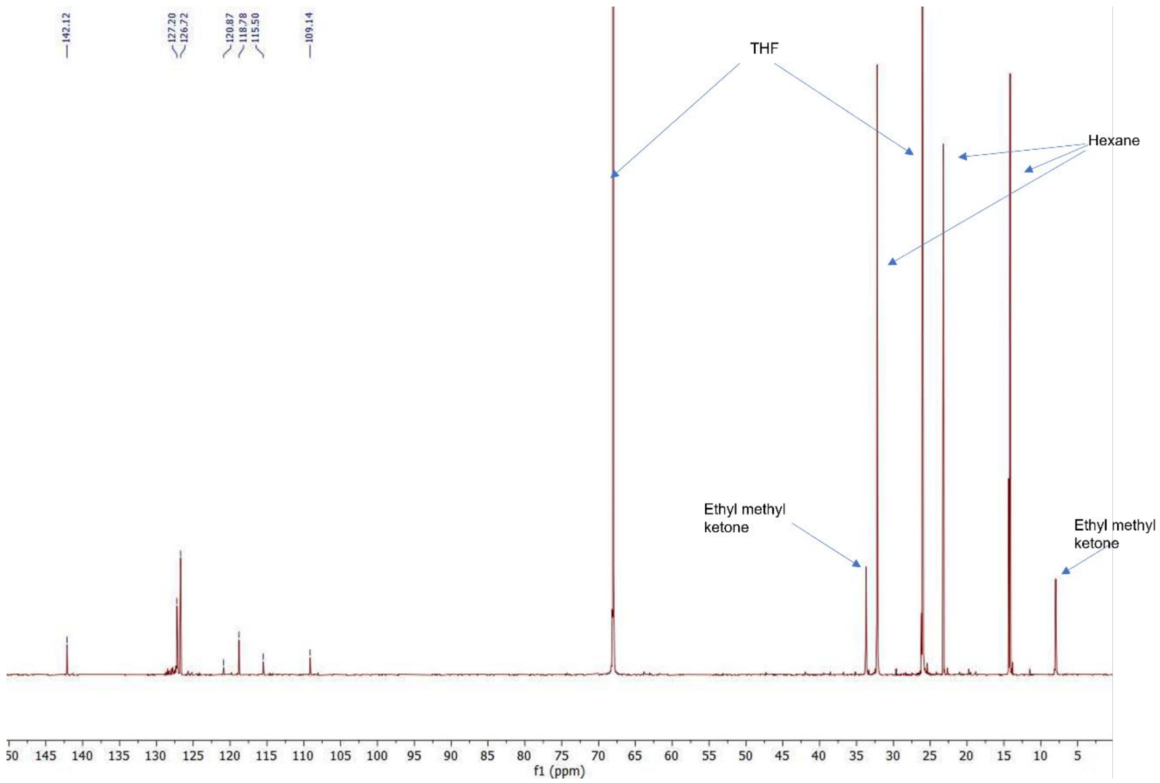

Figure S5: 126 MHz $^{13}C\{^{1}H\}$ NMR of $[Mg(THF)_3][Ph_4Pn]$ in THF-H_8

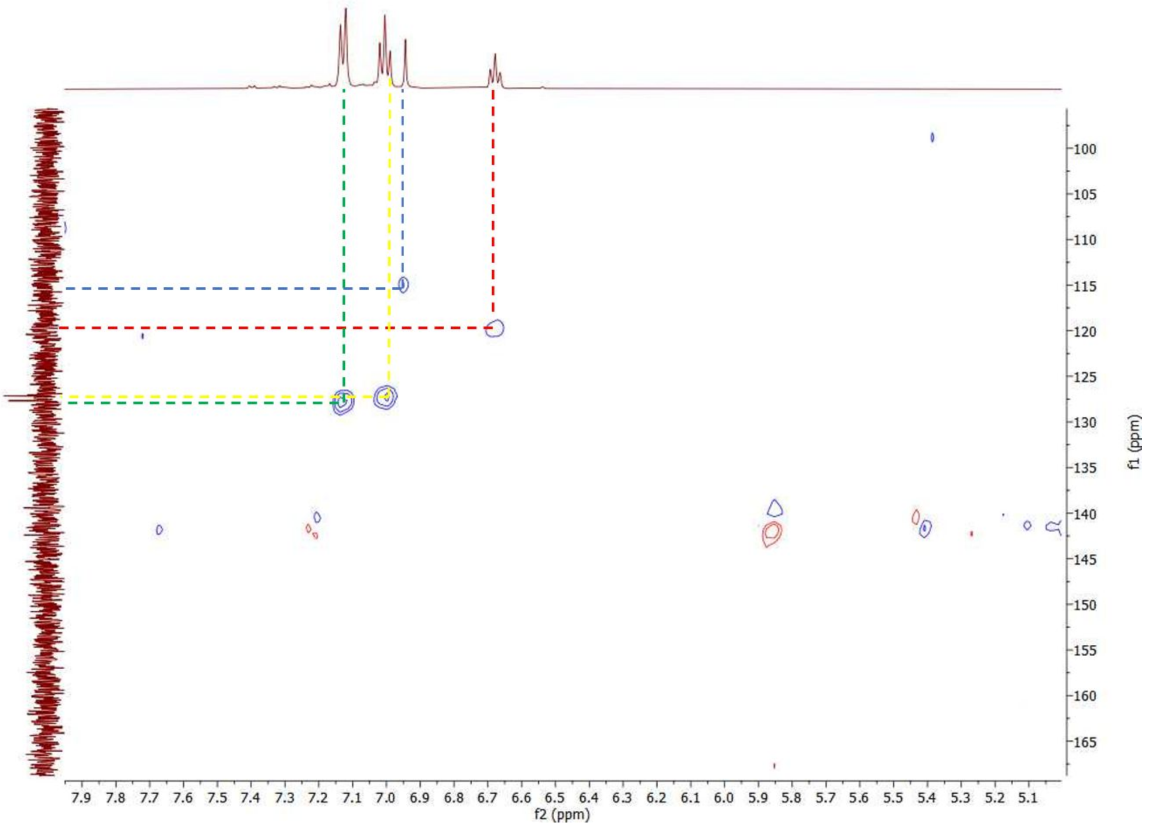

Figure S6: 500 MHz ^{1}H-^{13}C HSQC spectrum of $[Mg(THF)_3][Ph_4Pn]$ in THF-H_8 (Blue = H_w, Green = H_m, Yellow = H_m, Red = H_p)

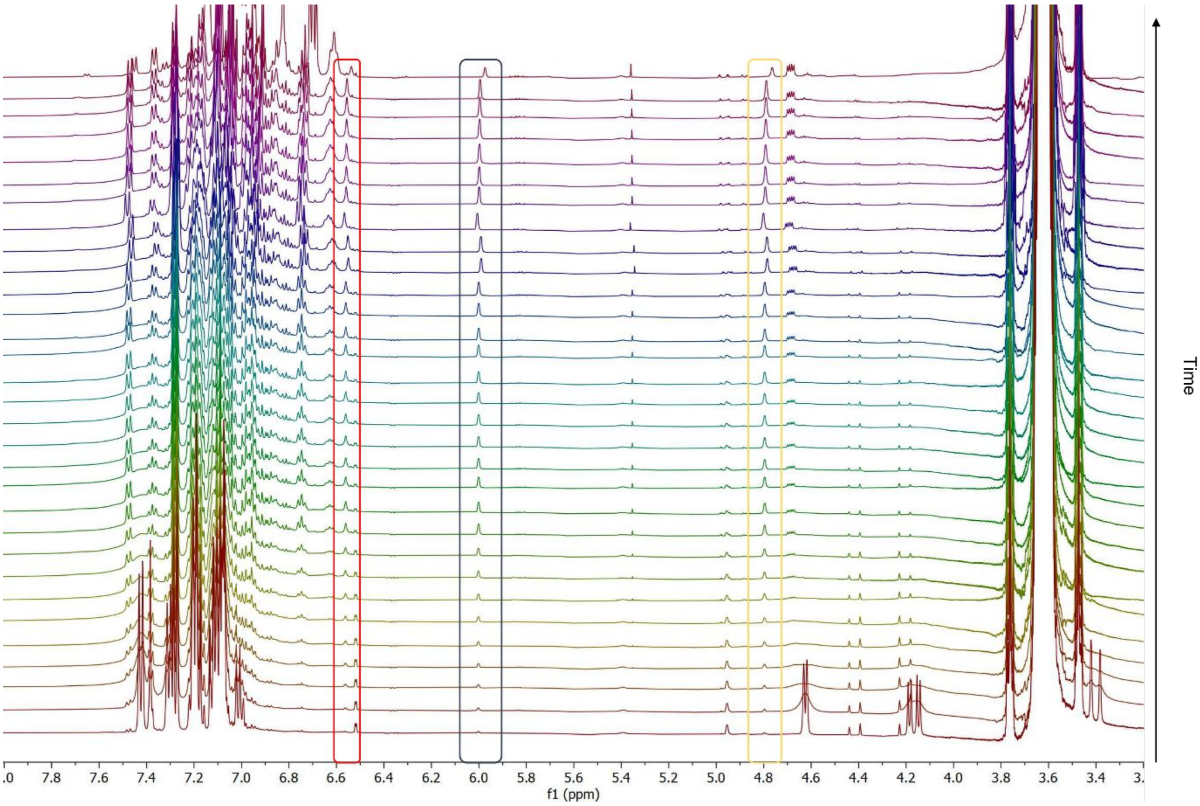

Figure S7: 500 MHz ^{1}H spectra of *in-situ* formation of [Mg(THF)$_3$][Ph$_4$Pn] in THF-H$_8$ over 48 hours. Highlighted resonances at 6.52 ppm, 5.96 ppm, 4.75 ppm show formation of hydropentalenide type intermediate.

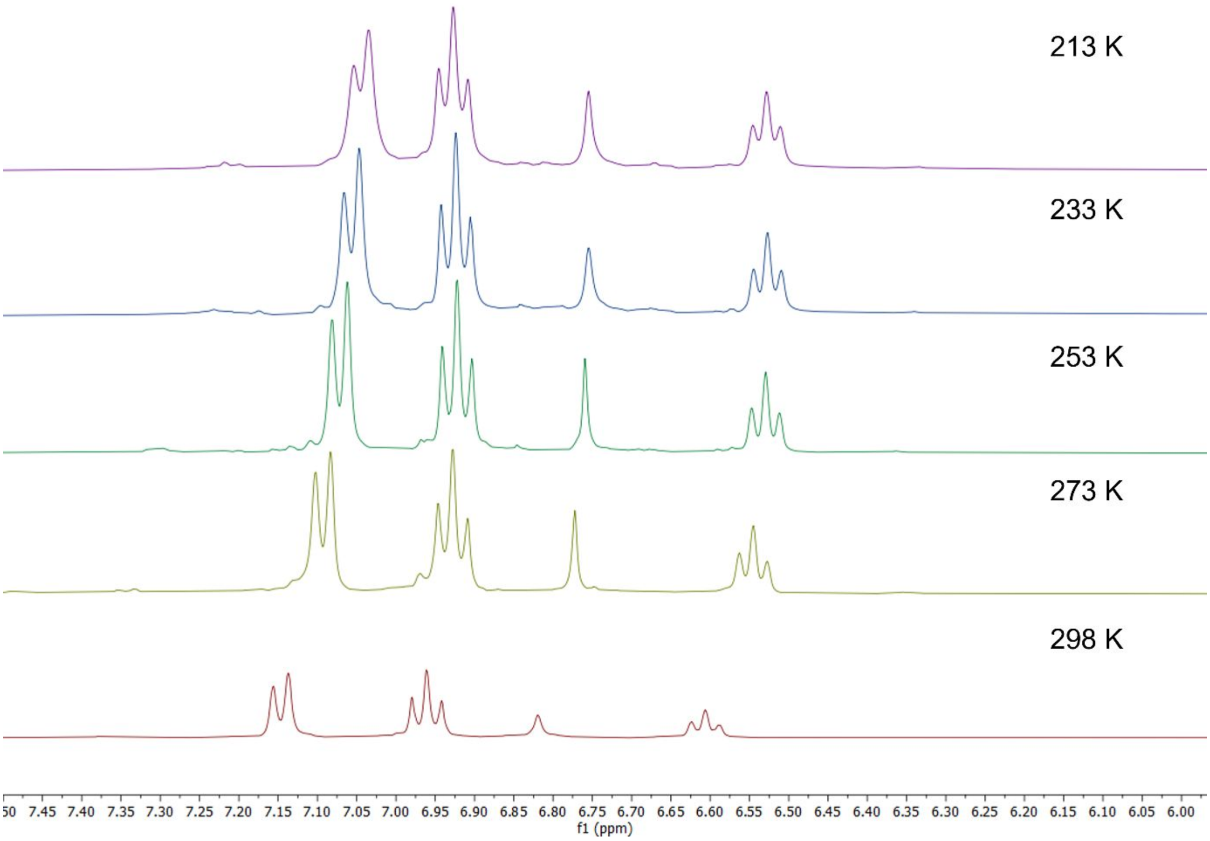

Figure S8: 400 MHz variable temperature ^1H NMR spectra of $[Mg(THF)_3][Ph_4Pn]$ in THF-H_8

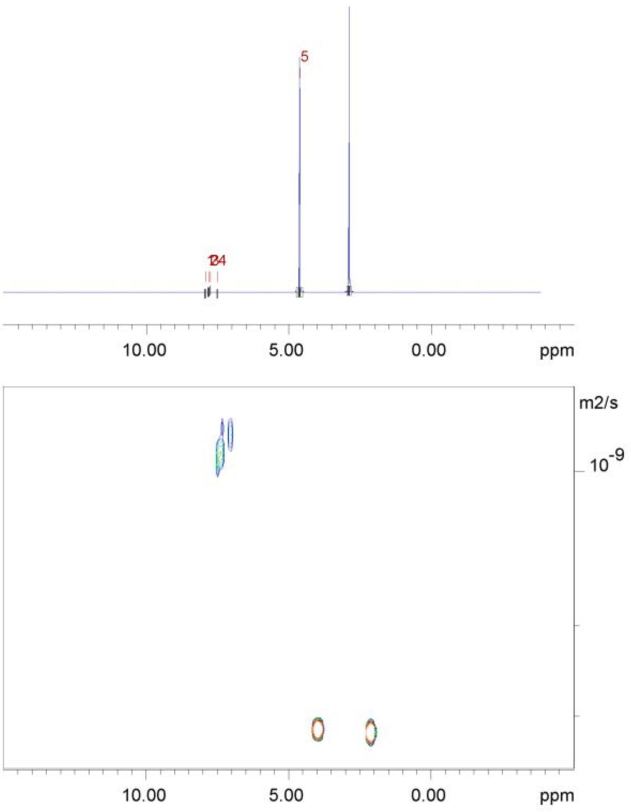

Peak name	F2 [ppm]	Io	error	D [m2/s]	error	fitInfo
1	7.475	1.31e+06	3.482e+04	9.41e-10	5.267e-11	Done
2	7.353	1.17e+06	2.796e+04	9.17e-10	4.603e-11	Done
3	7.289	2.94e+05	8225	8.30e-10	4.923e-11	Done
4	7.027	5.03e+05	1.634e+04	8.51e-10	5.836e-11	Done
5	3.969	3.71e+09	3.221e+07	3.20e-09	5.290e-11	Done
6	2.126	3.83e+09	3.619e+07	3.21e-09	5.767e-11	Done

Figure S9: 500 MHz ^{1}H DOSY NMR Spectrum (top) and experimentally determined diffusion coefficients (bottom) of $[Mg(THF)_3][Ph_4Pn]$ in THF-H_8

Di(butylmagnesium) 1,3,4,6-tetrapenhylpentalenide [Mg(^{n}Bu)(THF)$_2$][Ph$_4$Pn] (3)

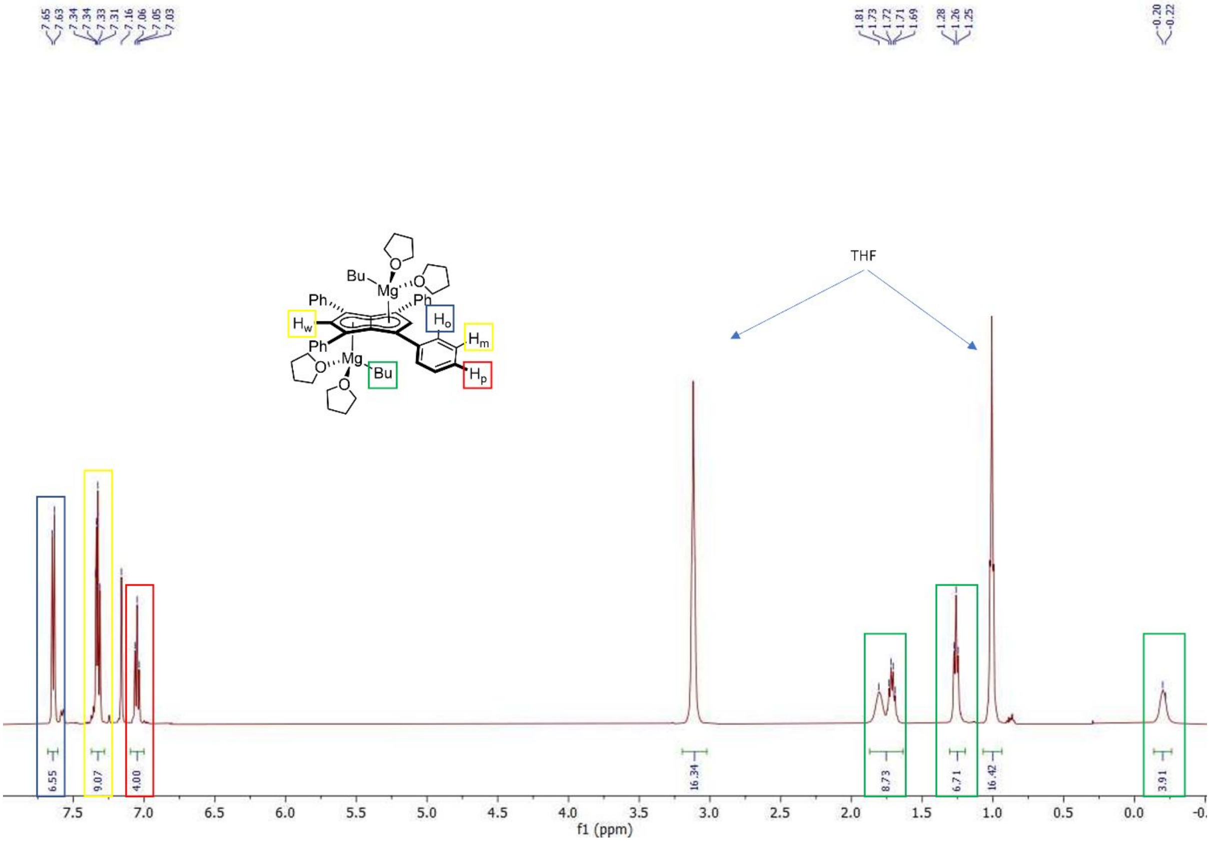

Figure S10: 500 MHz ^{1}H NMR of [MgBu(THF)$_2$]$_2$[Ph$_4$Pn] in C_6D_6

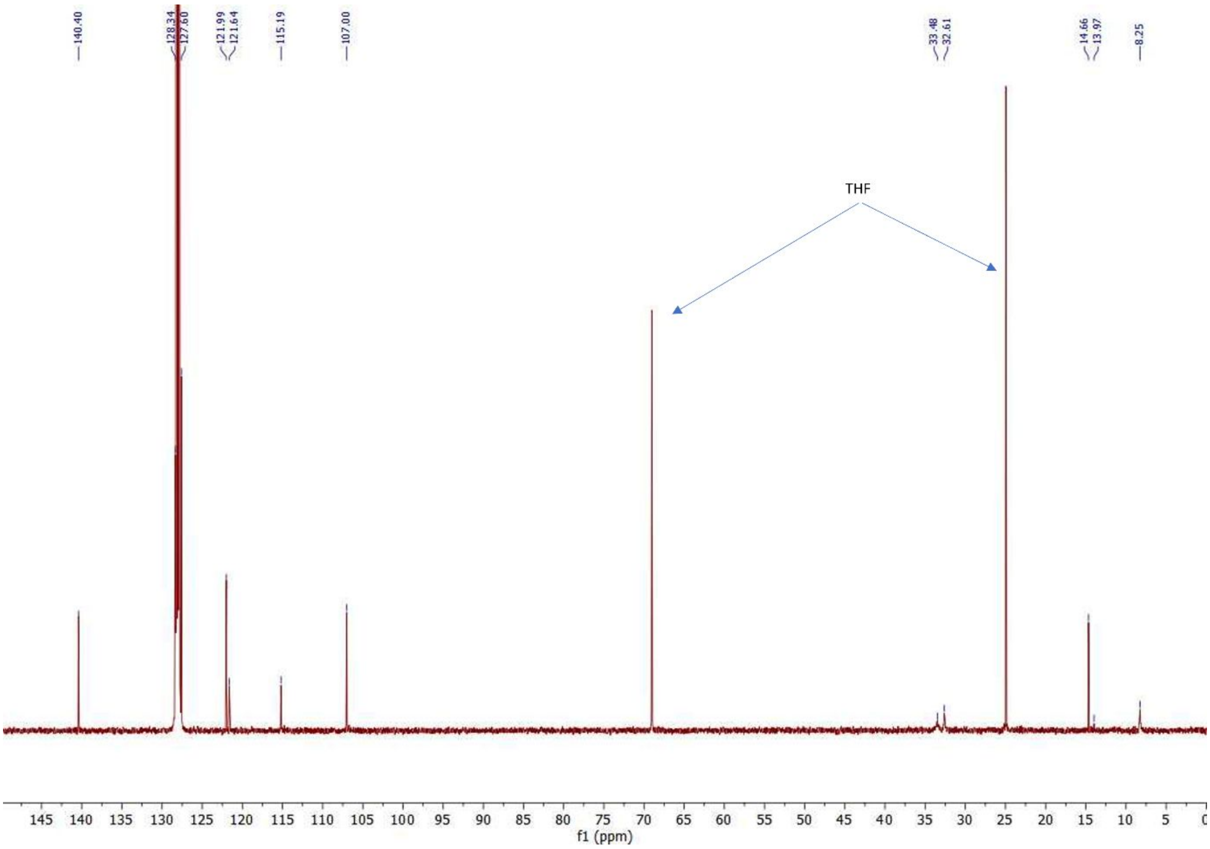

Figure S11: 126 MHz $^{13}C\{^{1}H\}$ NMR of $[MgBu(THF)_2]_2[Ph_4Pn]$ in C_6D_6

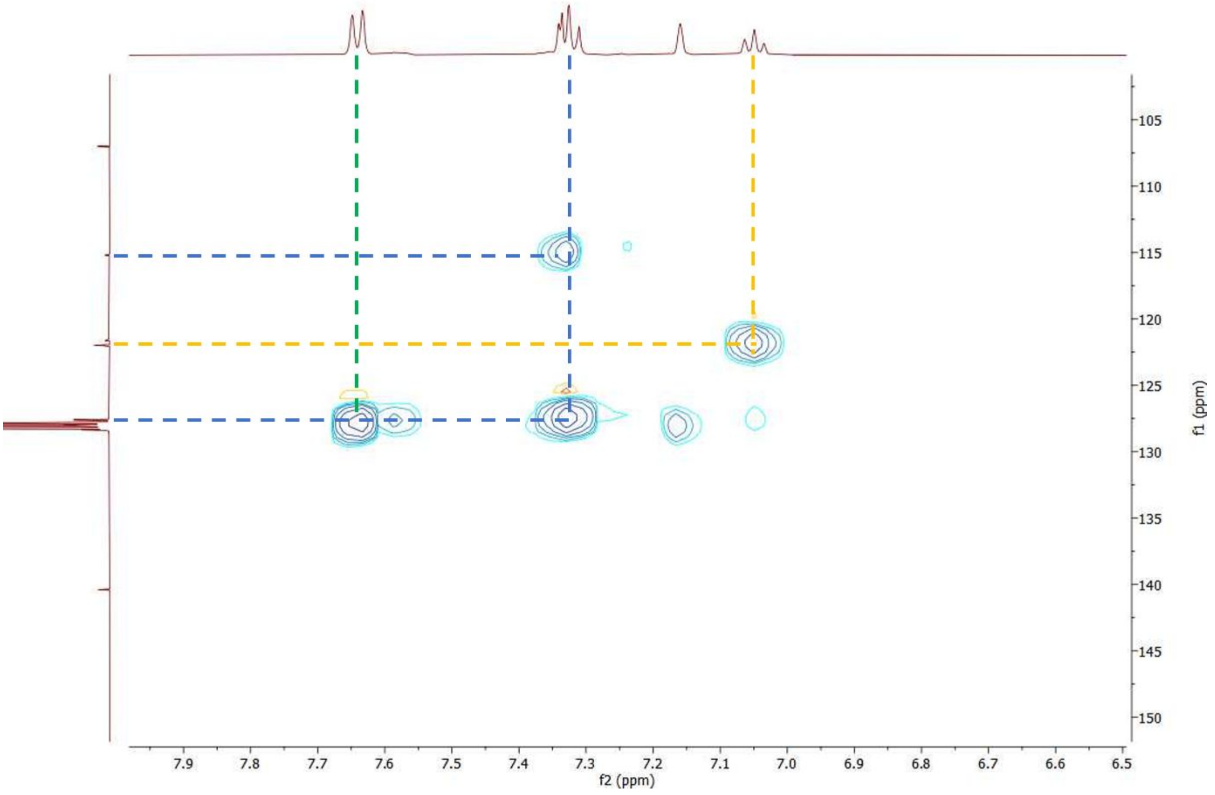

Figure S12: 500 MHz ^{1}H-^{13}C HSQC spectrum of $[MgBu(THF)_2]_2[Ph_4Pn]$ in C_6D_6 (Blue = H_m and H_w, Green = H_o, Yellow = H_p)

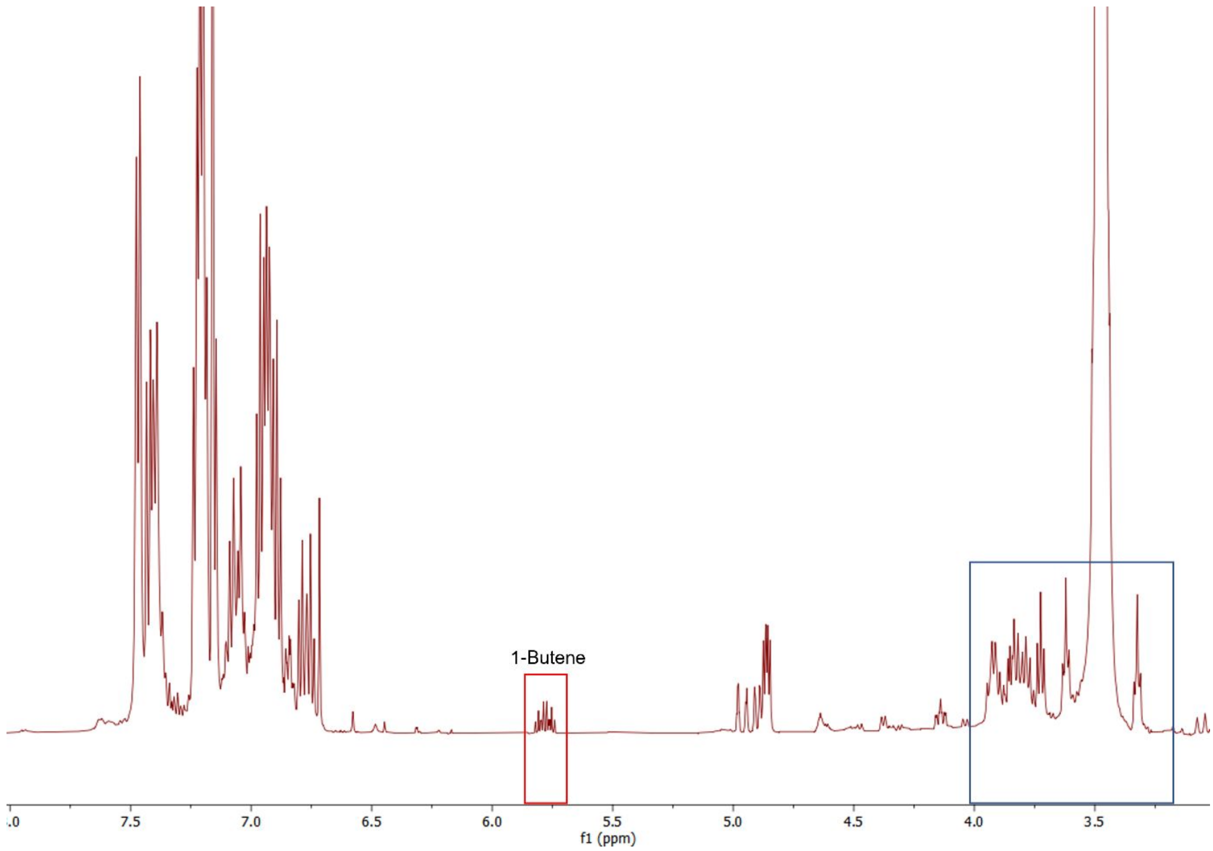

Figure S13: 500 MHz ^{1}H NMR spectrum of *in-situ* formation of $[MgBu(THF)_2]_2[Ph_4Pn]$ in C_6D_6 after one week

Commercial Dibutlymagnesium (1M in heptanes)

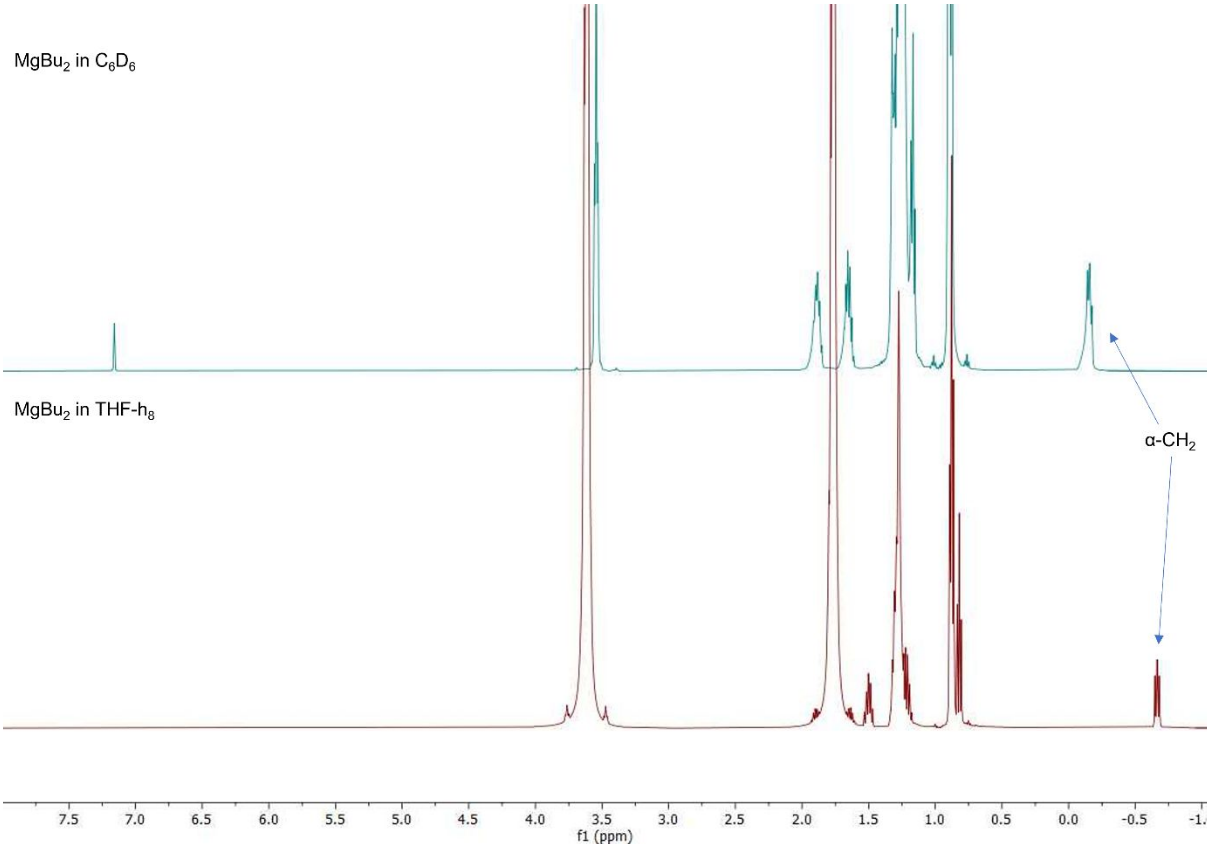

Figure S14: 500 MHz ^{1}H NMR spectra of $MgBu_2$ in C_6D_6 (top) and in THF-H_8 (bottom). ^{1}H α-CH_2 shifts = -0.15 ppm (C_6D_6) and -0.67 ppm (THF-H_8)

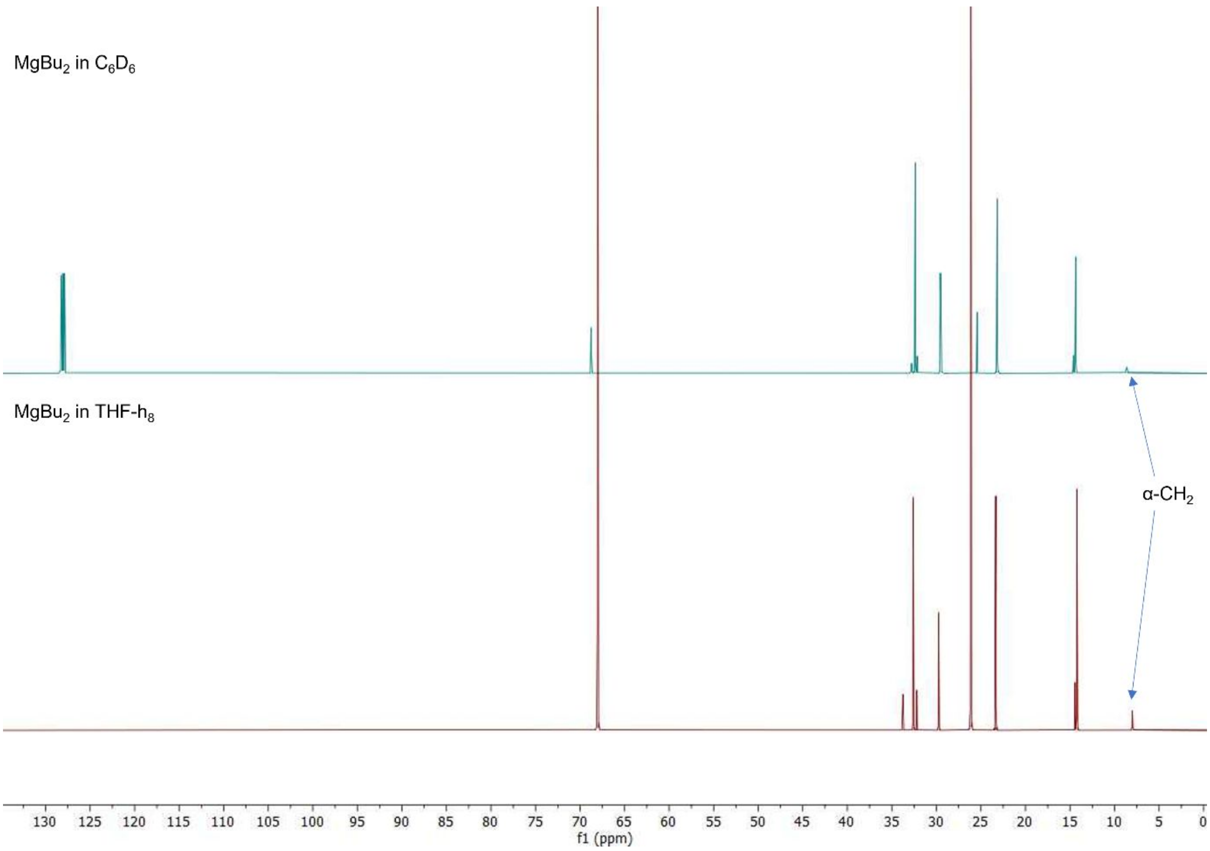

Figure S15: 126 MHz $^{13}C\{^1H\}$ NMR spectra of $MgBu_2$ in C_6D_6 (top) and in THF-H_8 (bottom). $^{13}C\{^1H\}$ α-CH_2 shifts = 8.6 ppm (C_6D_6) and 8.0 ppm (THF-H_8)

Interconversion Between [Mg(THF)$_3$][Ph$_4$Pn] and [Mg(^{n}Bu)(THF)$_2$]$_2$[Ph$_4$Pn]

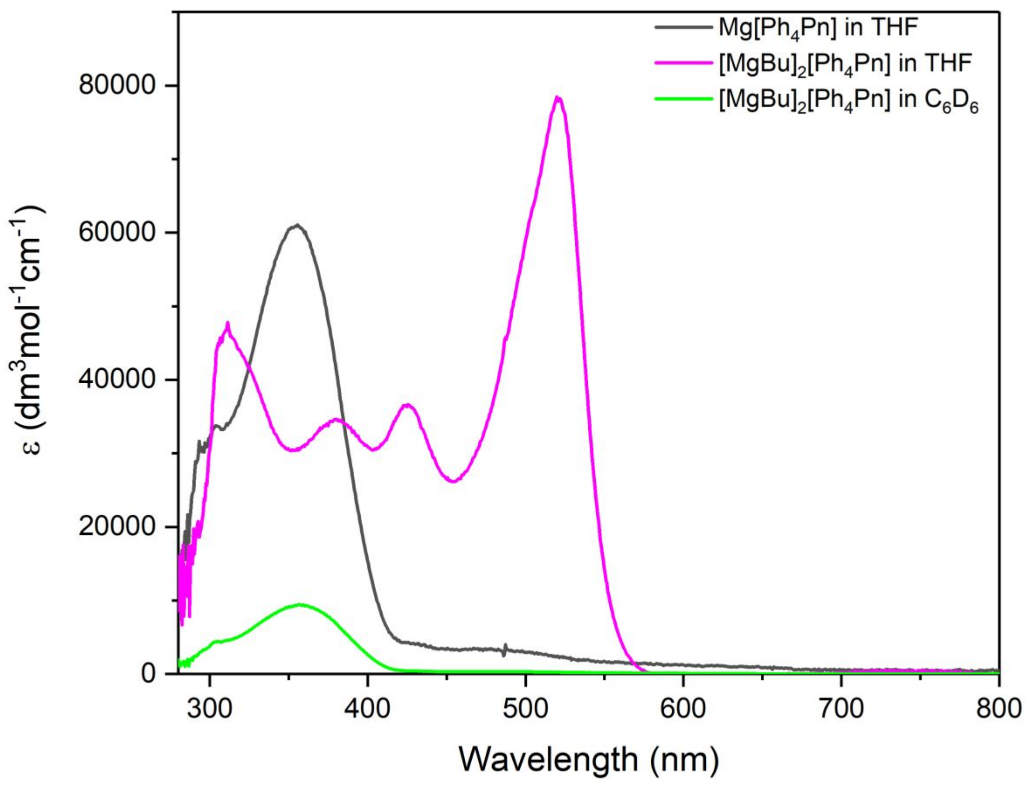

Figure S16: UV-vis spectrum of [Mg(THF)$_3$][Ph$_4$Pn] (1.5 x 10^{-5} M in THF), and [MgBu(THF)$_2$]$_2$[Ph$_4$Pn] (1 x 10^{-4} M in C_6D_6 and 1 x 10^{-5} M in THF) recorded at 298 K

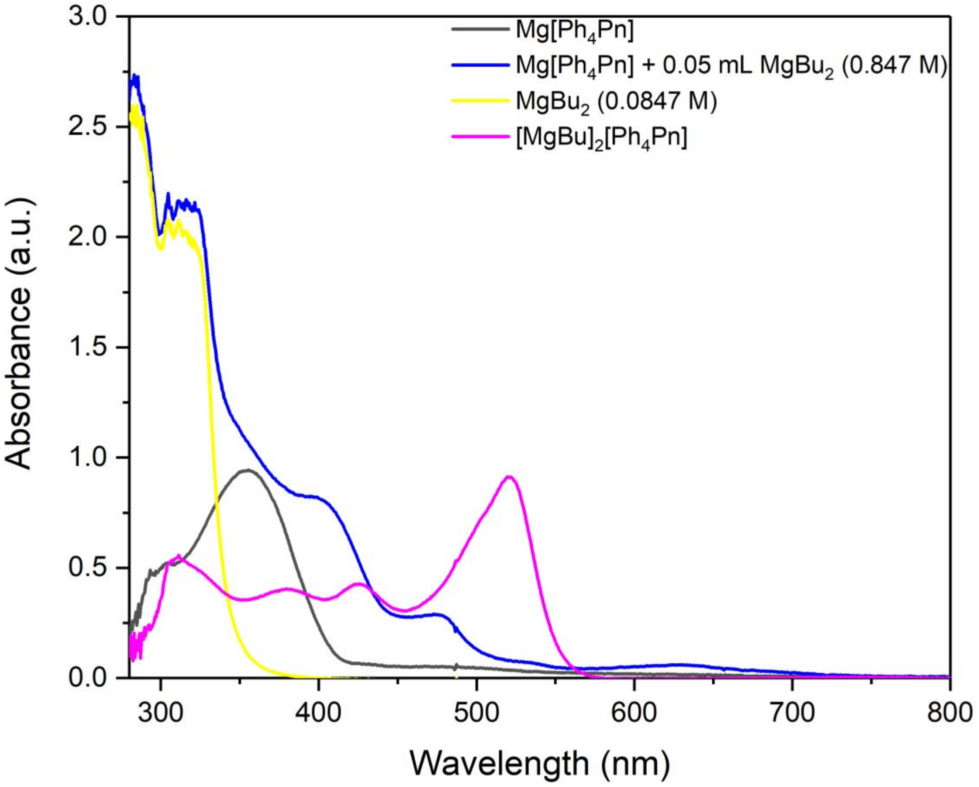

Figure S17: UV-vis spectrum of [Mg(THF)$_3$][Ph$_4$Pn] (1.5 x 10^{-5} M), [Mg(THF)$_3$][Ph$_4$Pn] (1.5 x 10^{-5} M) spiked with $MgBu_2$ (0.02 M) and [MgBu(THF)$_2$]$_2$[Ph$_4$Pn] (1.2 x 10^{-5} M) recorded in THF at 298 K.

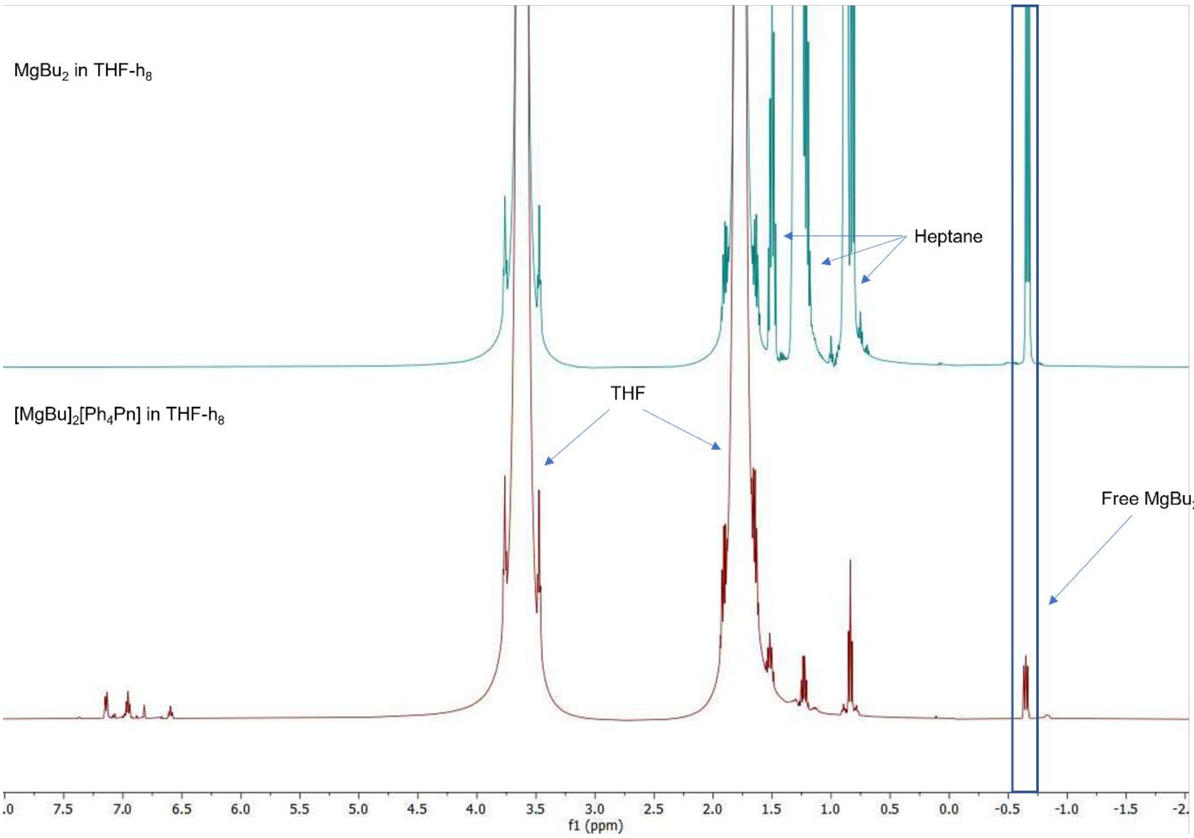

Figure S18: 500 MHz ^{1}H NMR spectra of $MgBu_2$ (top) and $[MgBu(THF)_2]_2[Ph_4Pn]$ in THF-H_8

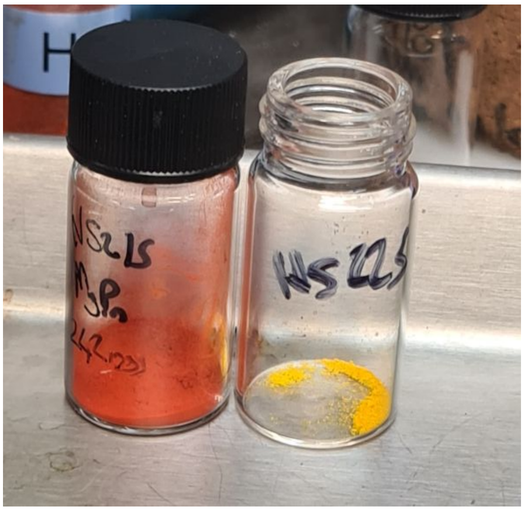

Figure S19: Photo of isolated $[Mg(THF)_3][Ph_4Pn]$ (left) and isolated $[MgBu(THF)_2]_2[Ph_4Pn]$ (right)

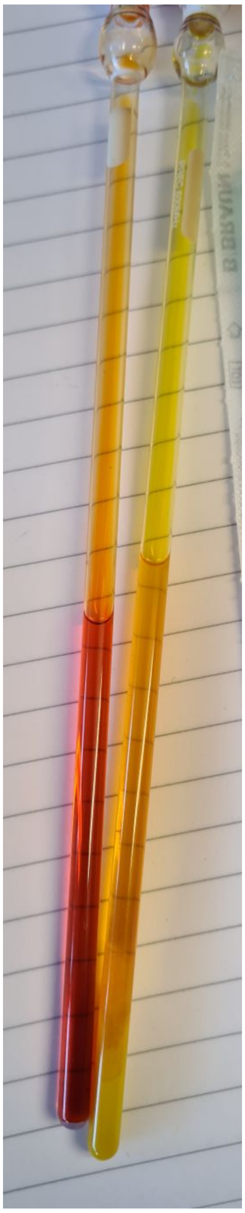

Figure S20: Photo of $[MgBu(THF)_2]_2[Ph_4Pn]$ in THF-H_8 (left) and $[MgBu(THF)_2]_2[Ph_4Pn]$ in C_6D_6 (right)

Butene Formation

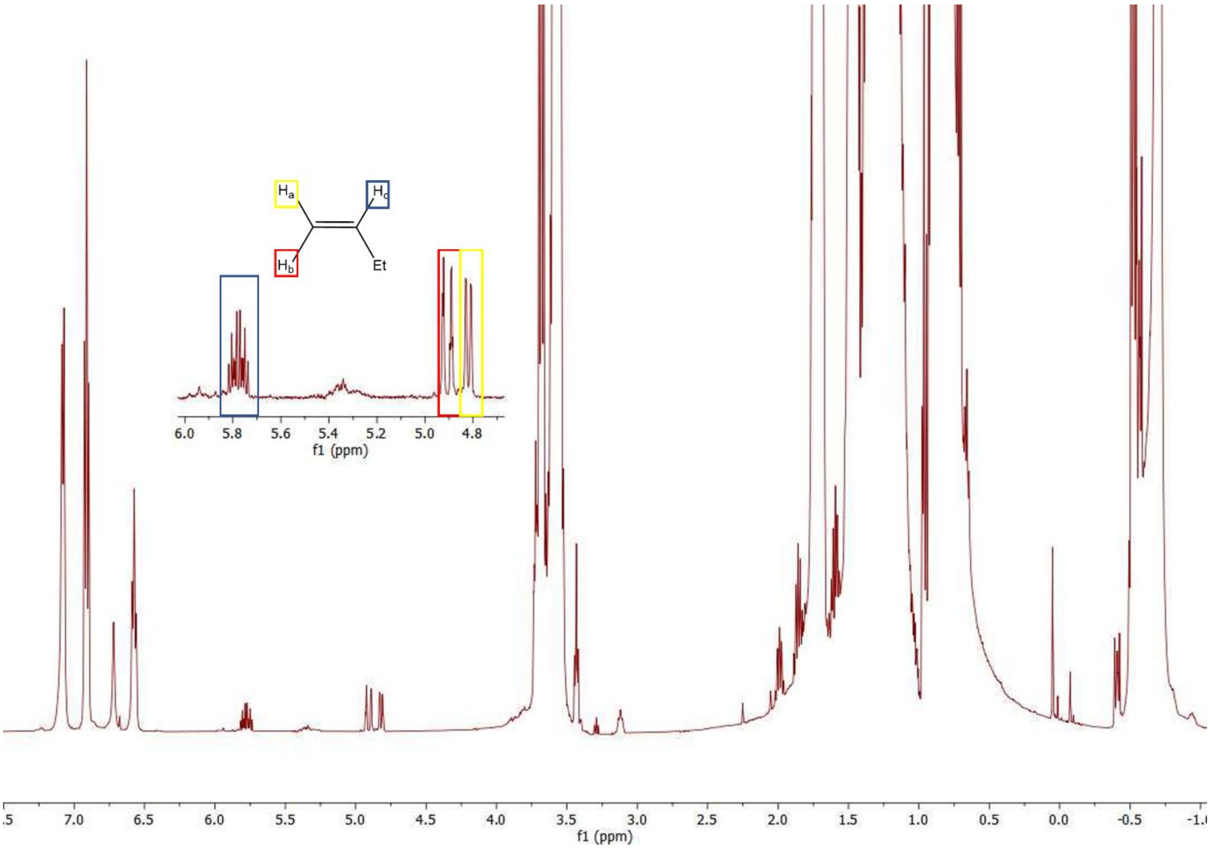

Figure S21: 500 MHz ^{1}H NMR spectrum of $[Mg(THF)_3][Ph_4Pn]$ and excess $MgBu_2$ showing 1-butene formation in THF-H_8

Hydrolysis of Magnesium 1,3,4,6-tetraphenylpentalenide

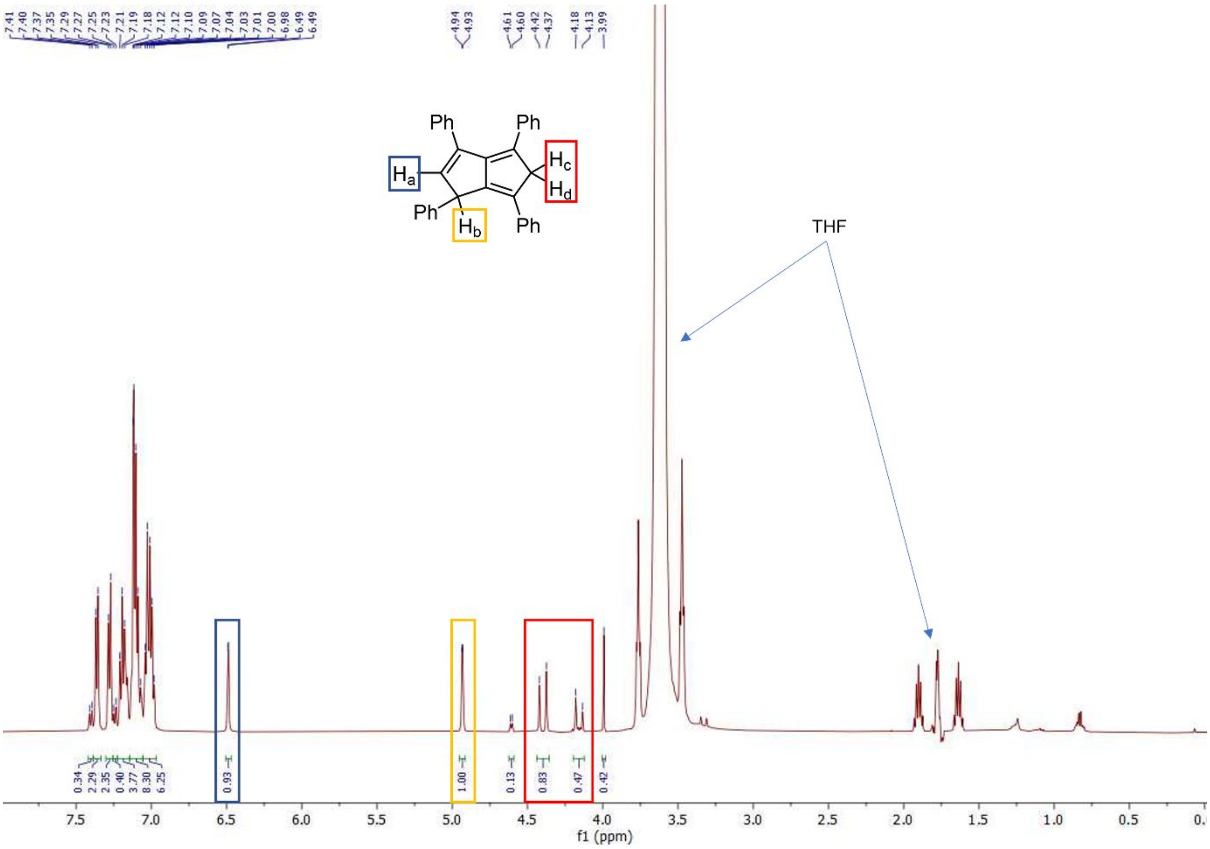

Figure S22: 500 MHz ^{1}H NMR spectrum of *in-situ* formed 1,3,4,6-Ph_4Pn-1,5-H_2 in THF-H_8. Spectrum obtained using the lc1gppnf2 solvent suppression pulse sequence, a double presaturation experiment during relaxation and mixing time using two independent channels.

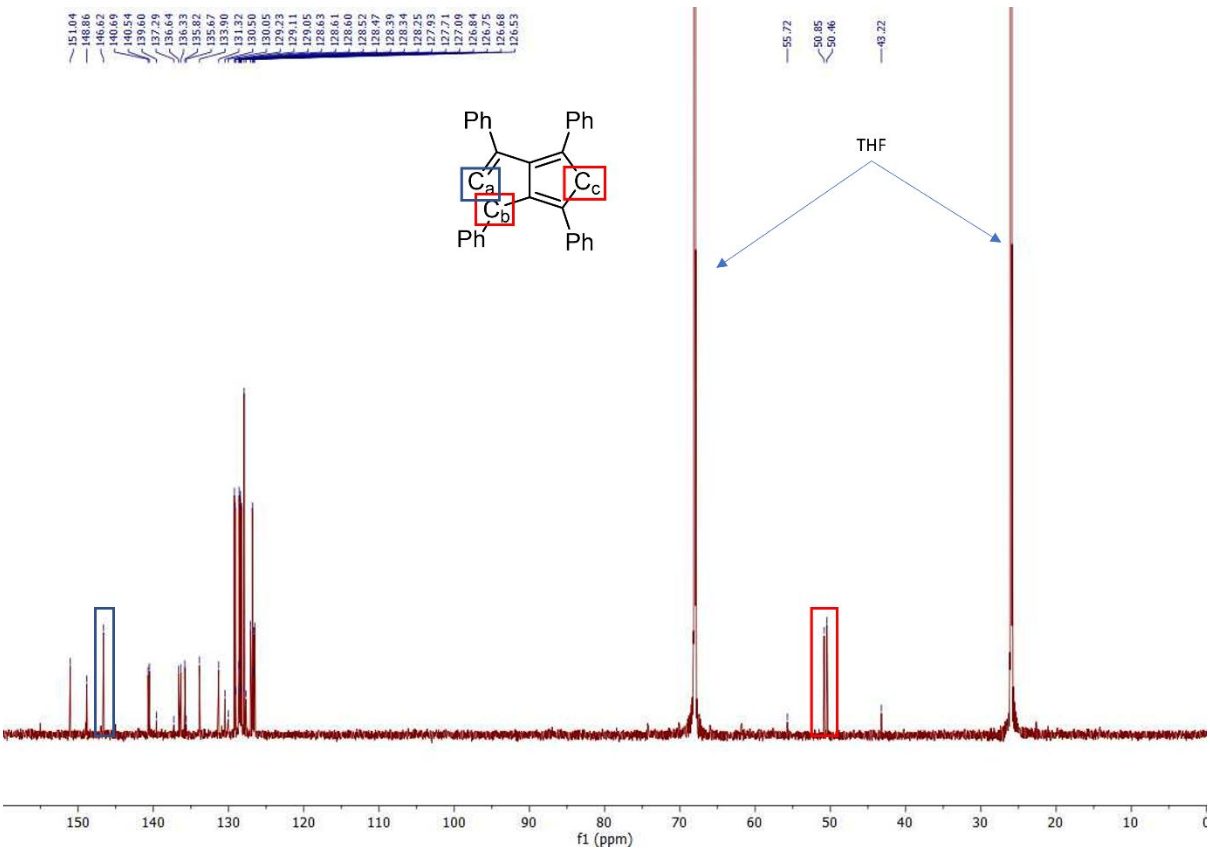

Figure S23: 126 MHz $^{13}C\{^1H\}$ NMR spectrum of *in-situ* formed 1,3,4,6-Ph_4Pn-1,5-H_2 in THF-H_8

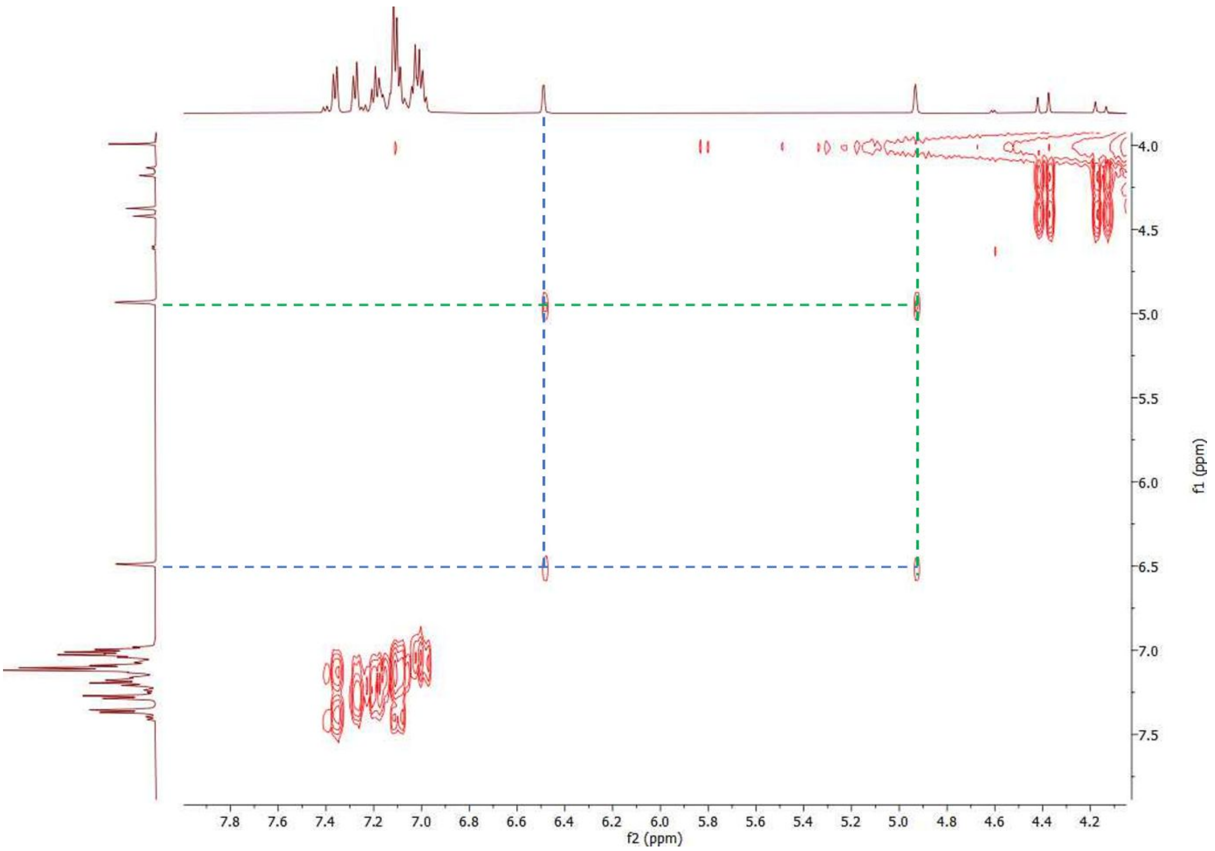

Figure S24: 500 MHz ^1H-^1H COSY spectrum of *in-situ* formed 1,3,4,6-Ph_4Pn-1,5-H_2 in THF-H_8 (Blue = H_a, Green = H_b)

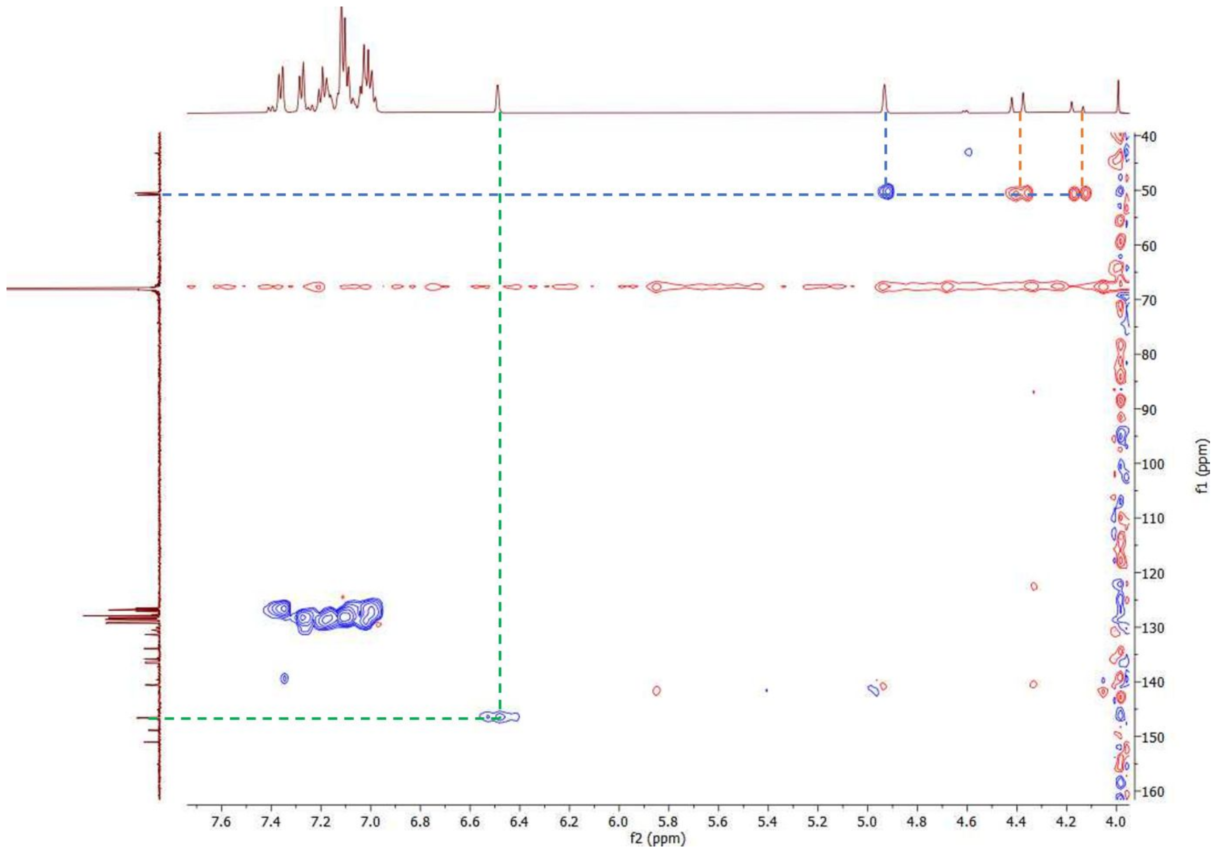

Figure S25: 500 MHz ^{1}H-^{13}C HSQC spectrum of *in-situ* formed 1,3,4,6-Ph_4Pn-1,5-H_2 in THF-H_8 (Blue = H_b, Orange = H_c/H_d, Green = H_a)

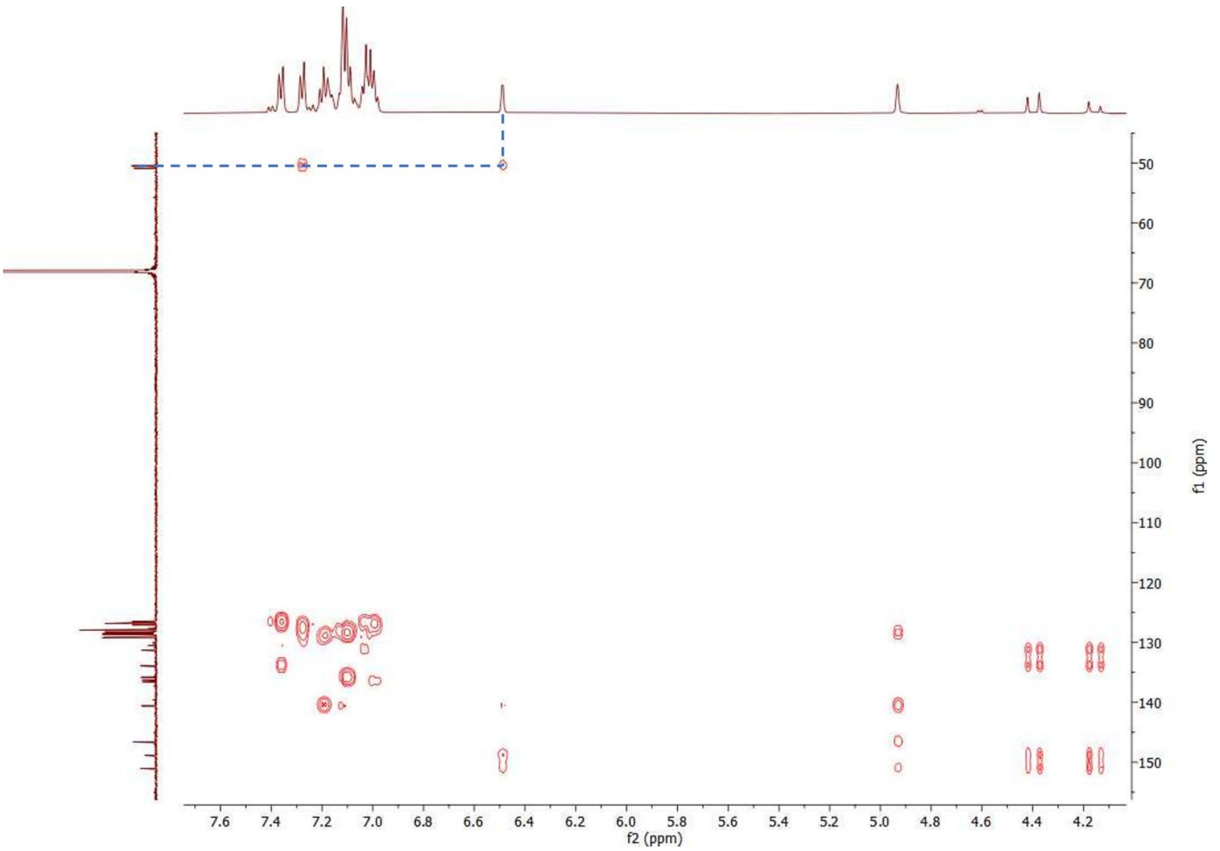

Figure S26: 500 MHz ^{1}H-^{13}C HMBC spectrum of *in-situ* formed 1,3,4,6-Ph_4Pn-1,5-H_2 in THF-H_8 (Blue = H_a)

Deuteration of Magnesium 1,3,4,6-tetraphenylpentalenide

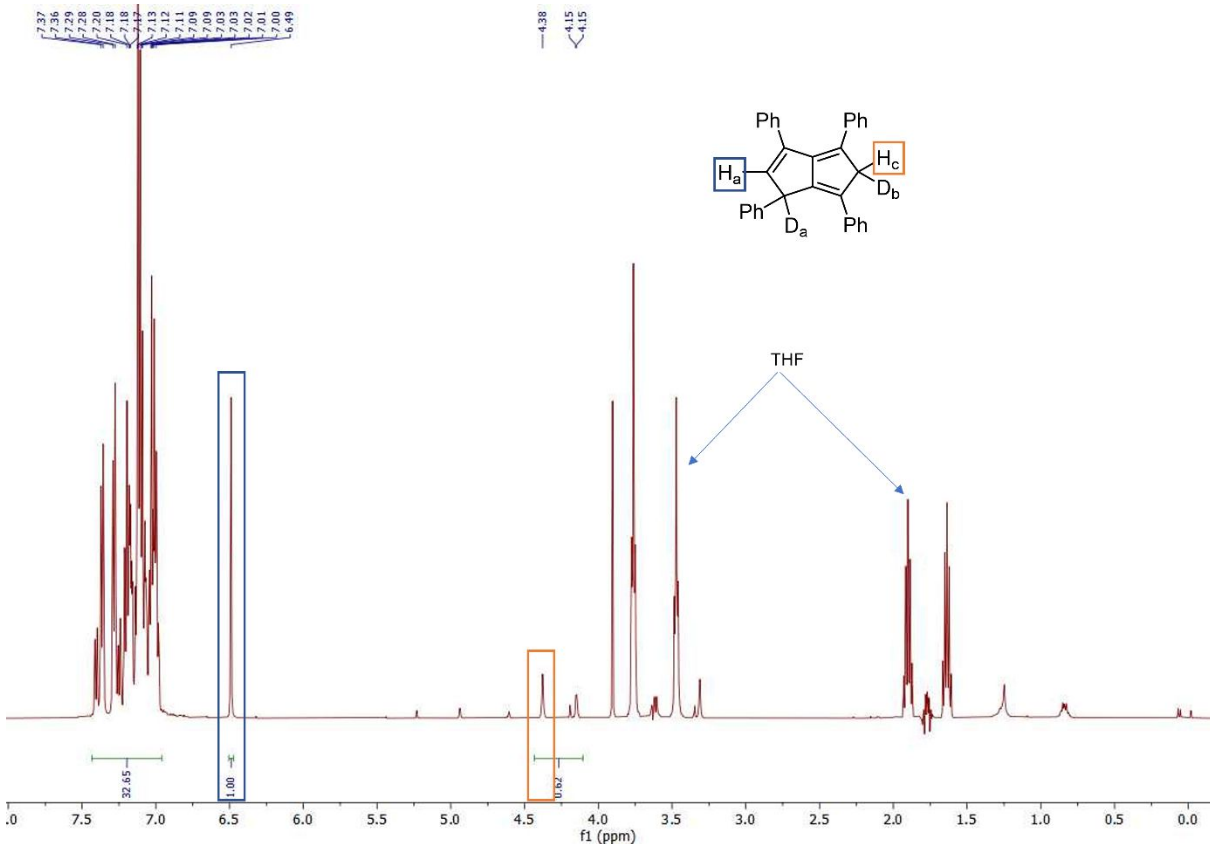

Figure S27: 500 MHz ^1H NMR spectrum of *in-situ* formed 1,3,4,6-Ph_4Pn-1,5-D_2 in THF-H_8. Spectrum obtained using the lc1gppnf2 solvent suppression pulse sequence, a double presaturation experiment during relaxation and mixing time using two independent channels.

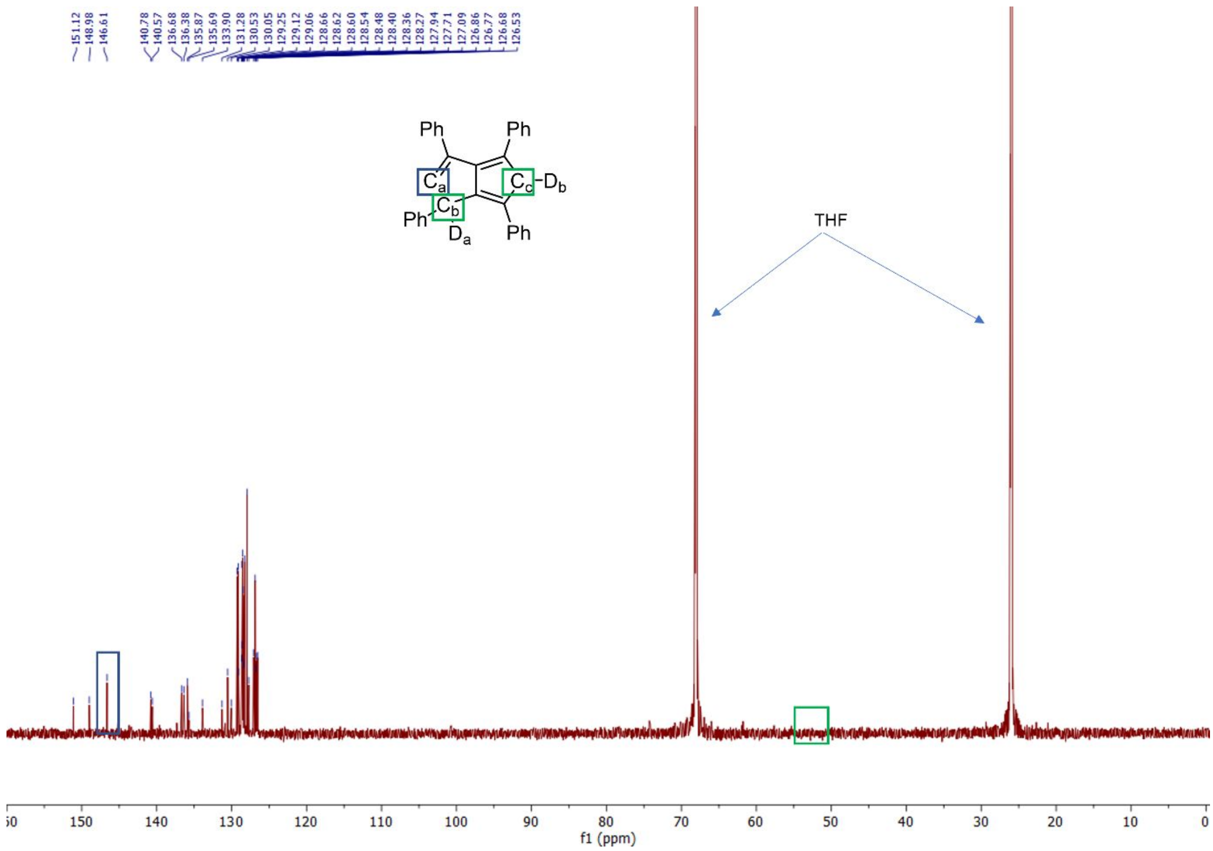

Figure S28: 126 MHz $^{13}C\{H\}$ NMR spectrum of *in*-situ formed 1,3,4,6-Ph_4Pn-1,5-D_2 in THF-H_8

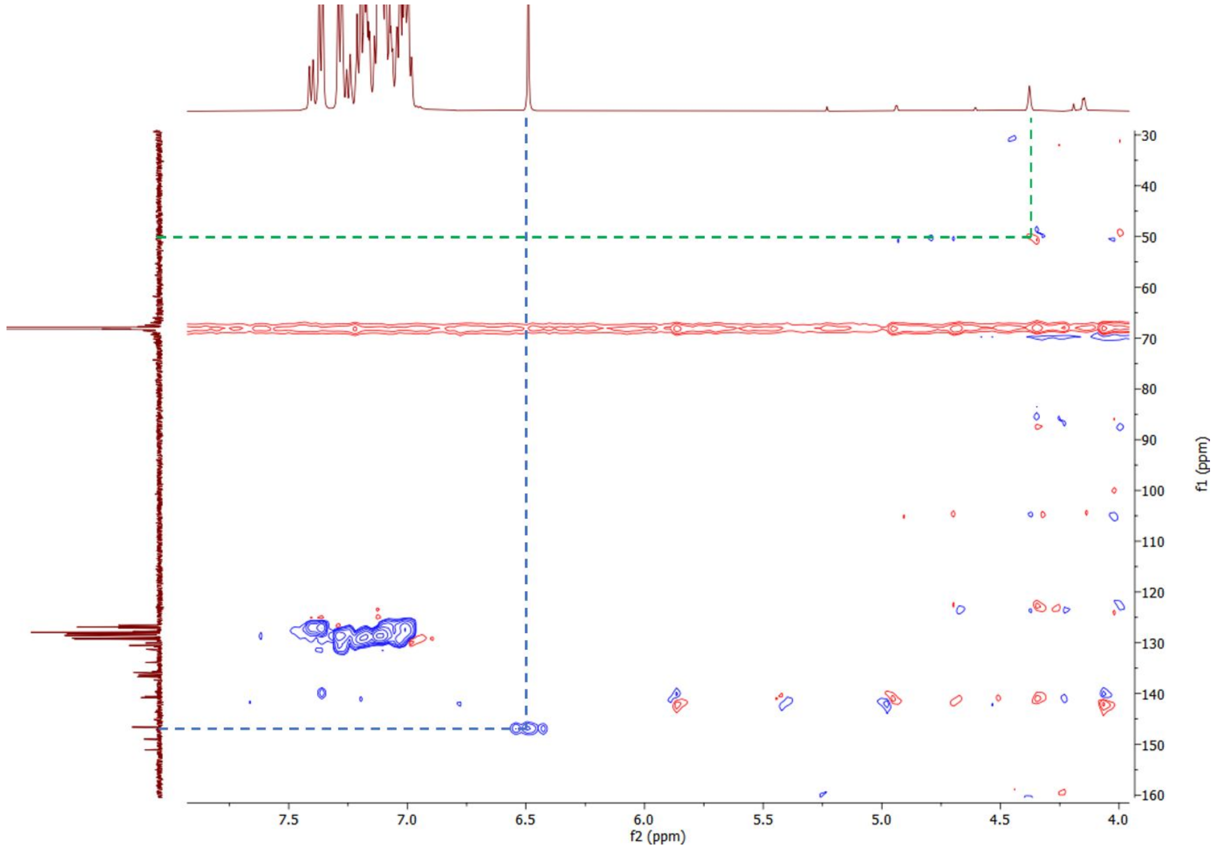

Figure S29: 500 MHz ^{1}H-^{13}C HSQC spectrum of *in-situ* formed 1,3,4,6-Ph_4Pn-1,5-D_2 in THF-H_8 (Blue = H_a, Green = H_c)

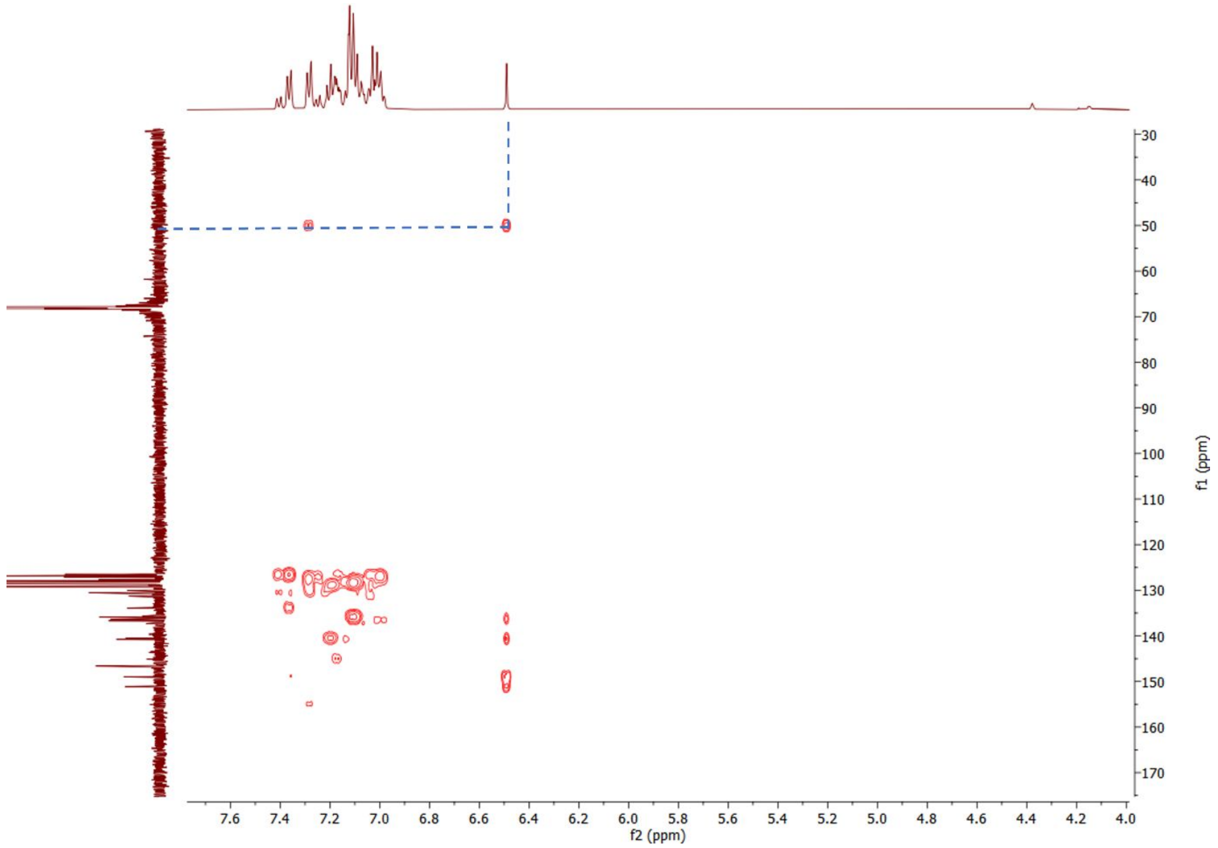

Figure S30: 500 MHz ^1H-^{13}C HMBC spectrum of *in-situ* formed 1,3,4,6-Ph_4Pn-1,5-D_2 in THF-H_8 (Blue = H_a)

1,5-dimethyl-1,3,4,6-tetraphenyl-5-hydropentalene

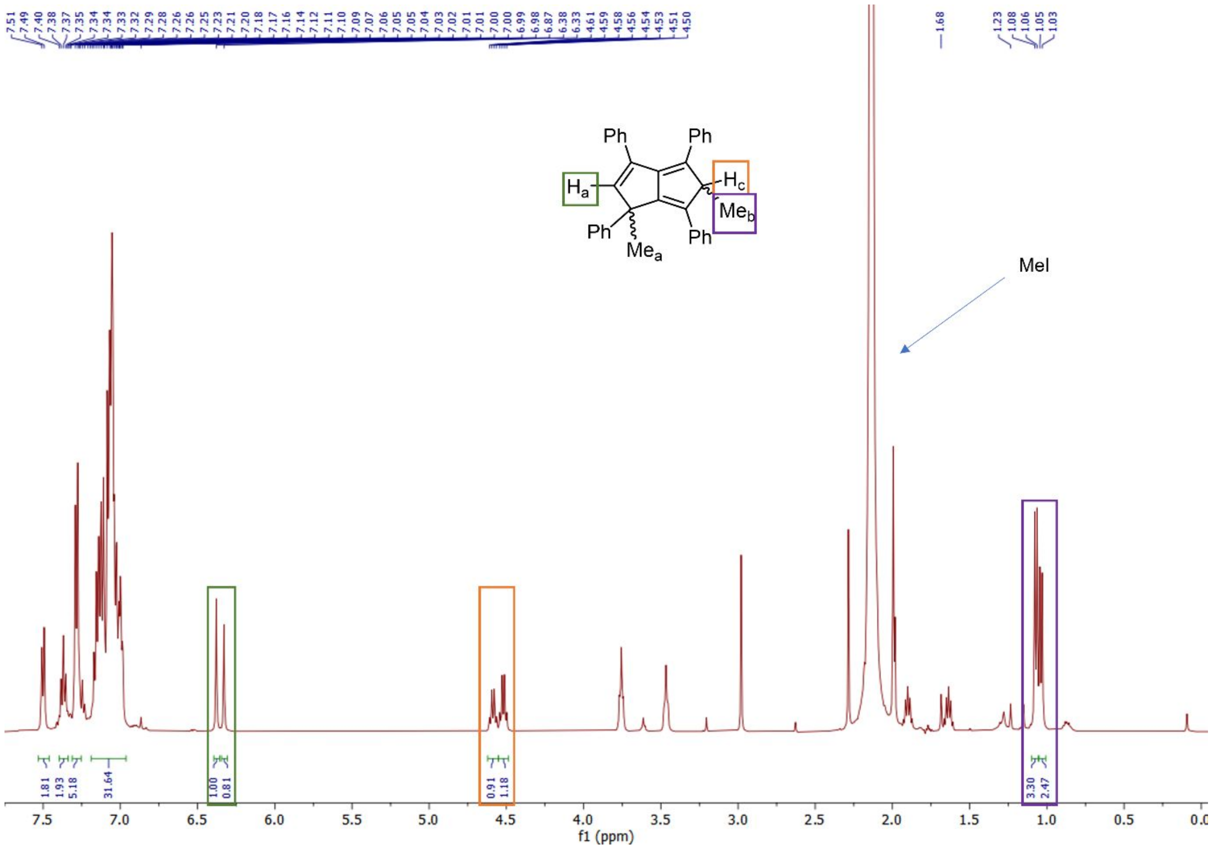

Figure S31: 500 MHz ^{1}H NMR spectrum of *in-situ* formed 1,3,4,6-Ph_4-1,5-Me_2PnH in THF-H_8. Spectrum obtained using the lc1gppnf2 solvent suppression pulse sequence, a double presaturation experiment during relaxation and mixing time using two independent channels.

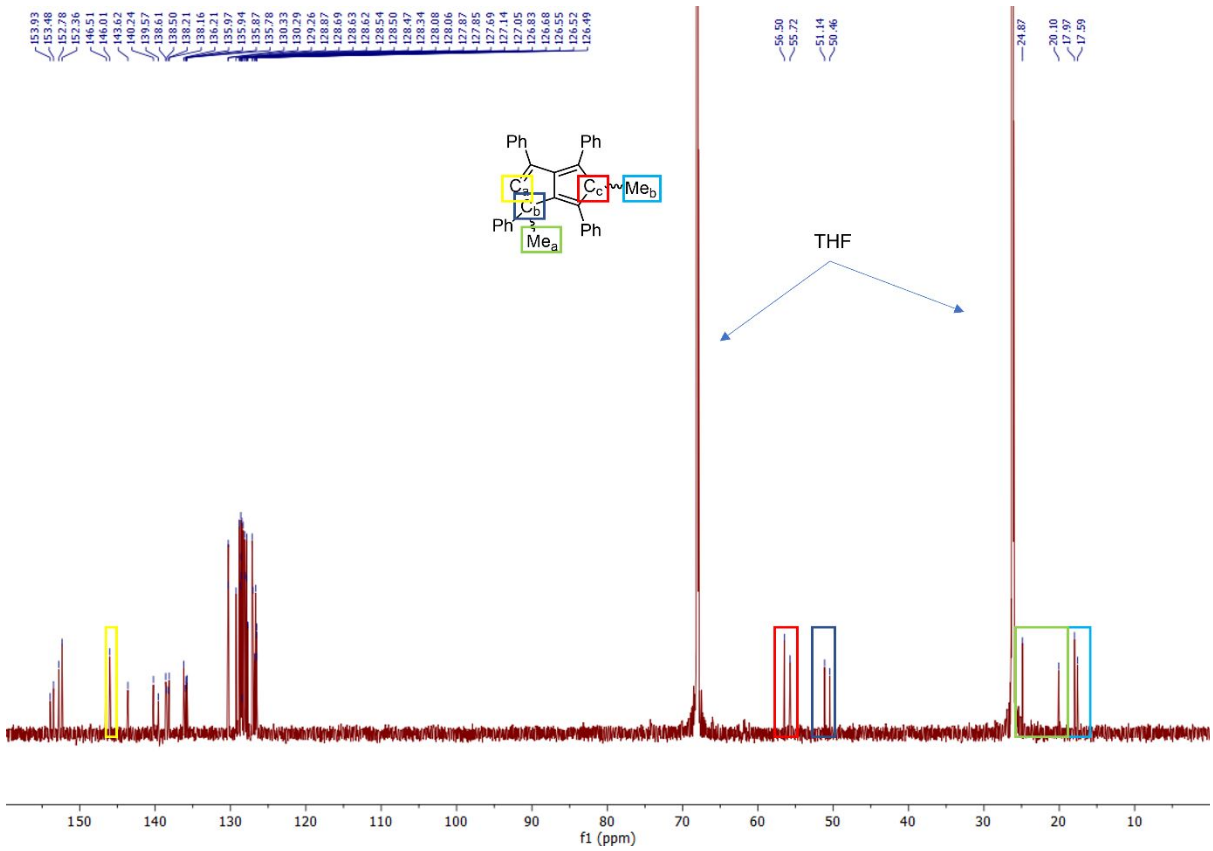

Figure S32: 126 MHZ $^{13}C\{^{1}H\}$ NMR spectrum of *in-situ* formed 1,3,4,6-Ph_4-1,5-Me_2PnH in THF-H_8

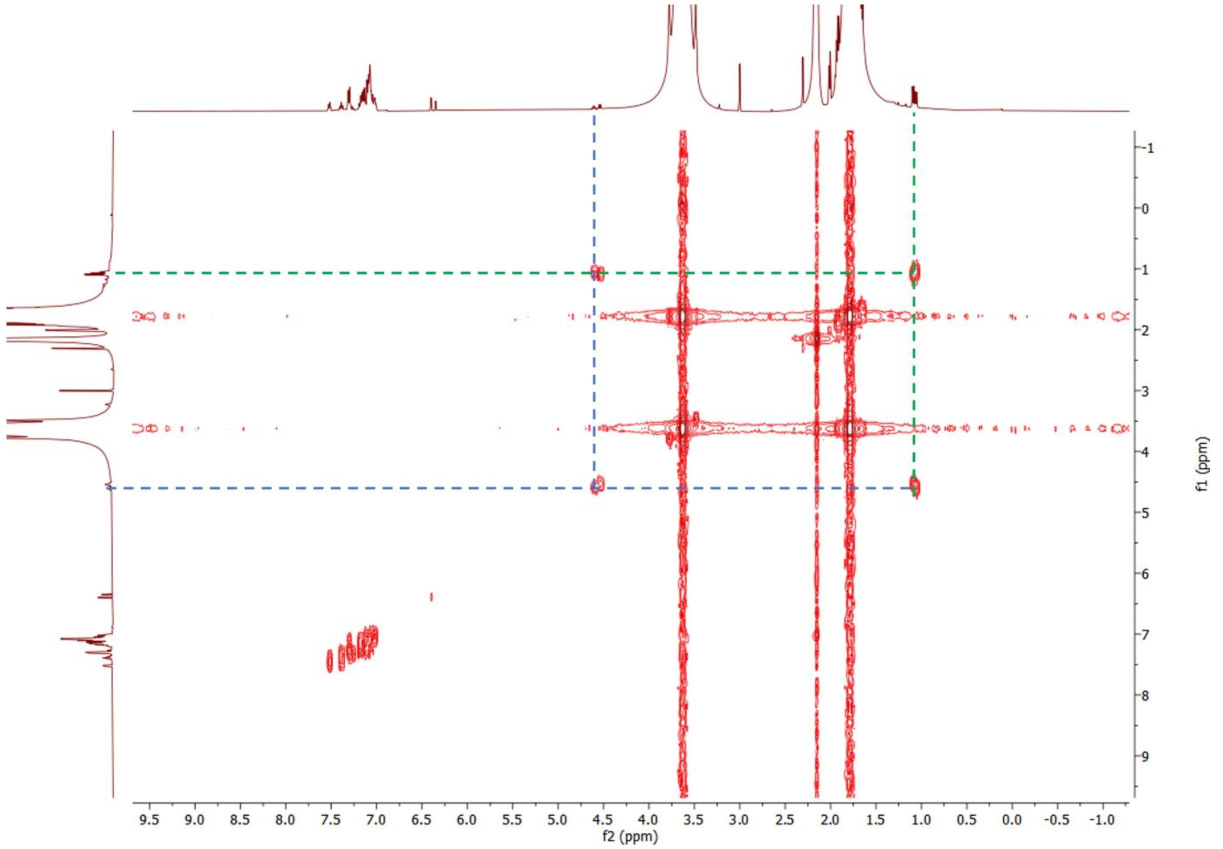

Figure S33: 500 MHz ^{1}H-^{1}H COSY spectrum of *in-situ* formed 1,3,4,6-Ph_4-1,5-Me_2PnH in THF-H_8 (Blue = H_c and Green = Me_b)

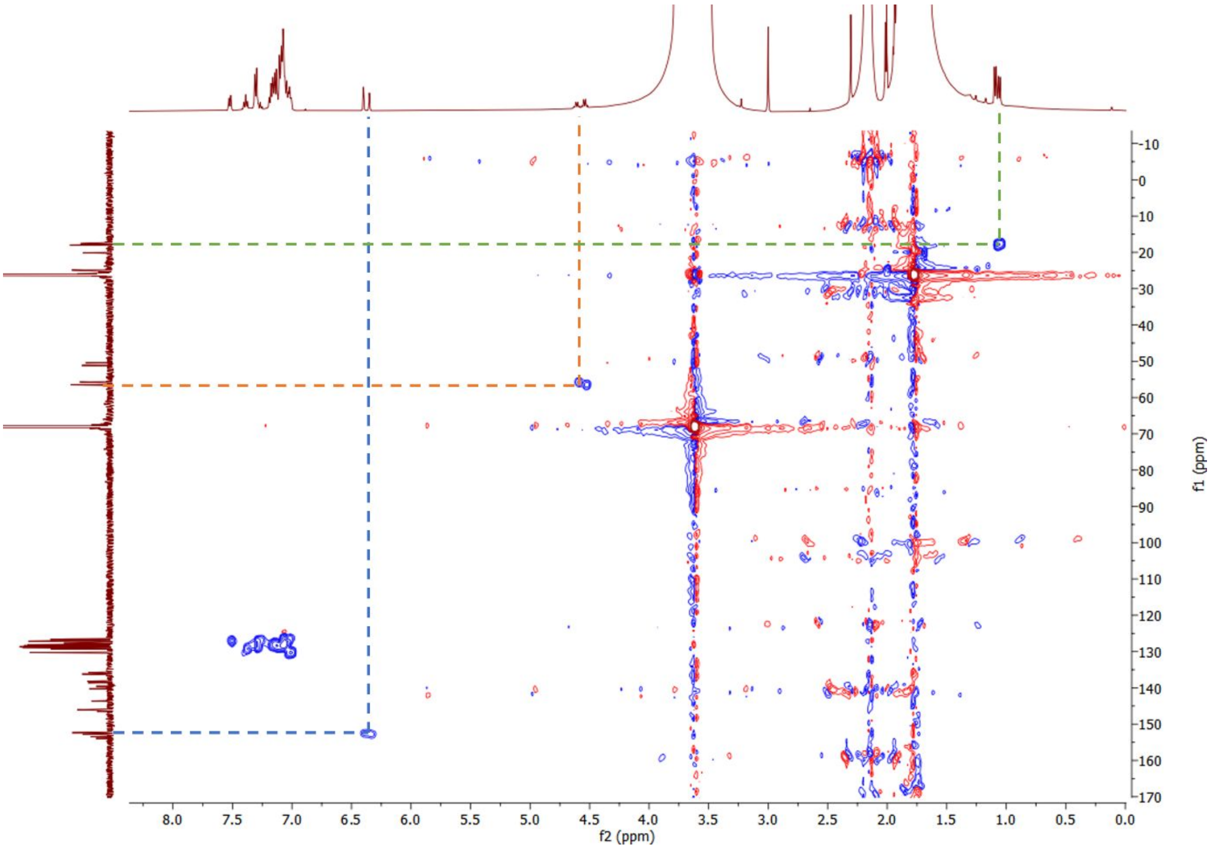

Figure S34: 500 MHz ^{1}H-^{13}C HSQC spectrum of *in-situ* formed 1,5-Me_2-1,3,4,6-Ph_4PnH in THF-H_8 (Blue = H_a, Orange = H_c, Green = Me_b)

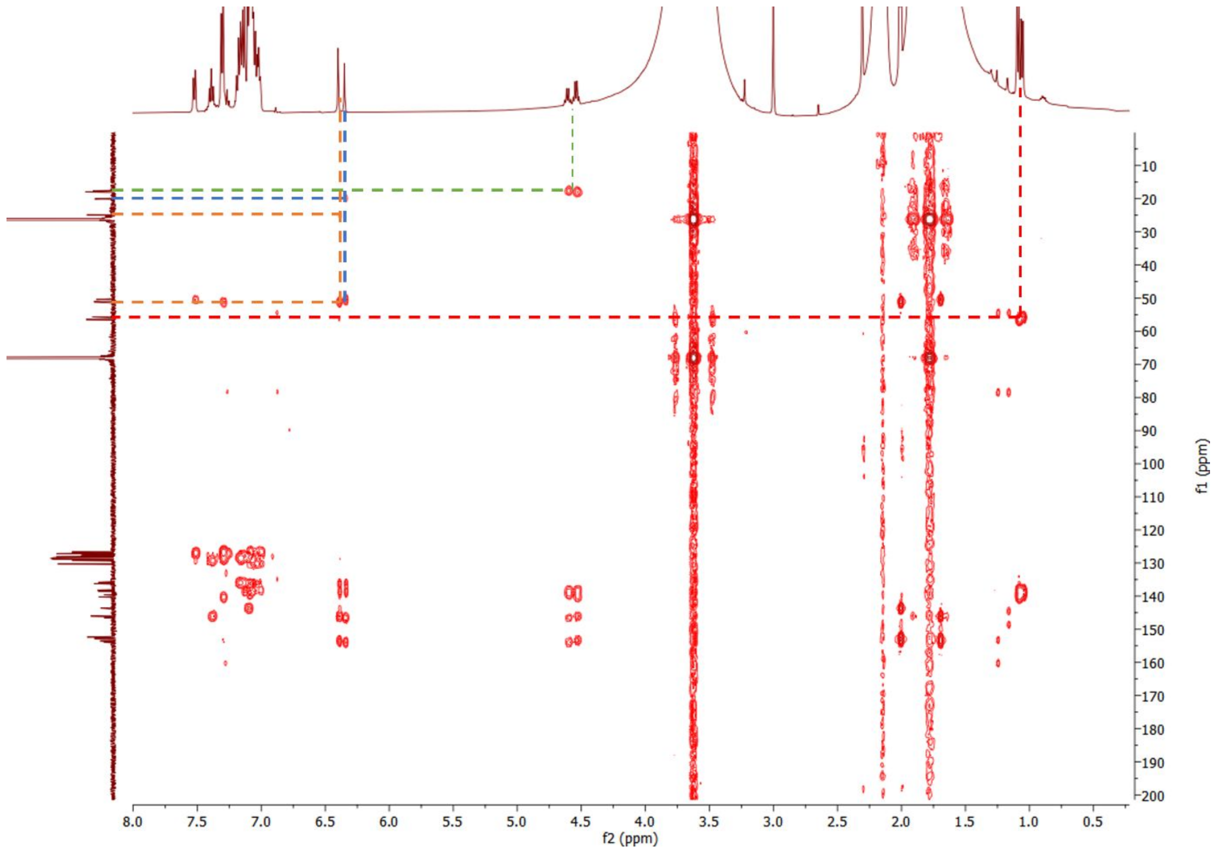

Figure S35: 500 MHz ^1H-^{13}C HMBC spectrum of *in-situ* formed 1,5-Me_2-1,3,4,6-Ph_4PnH in THF-H_8 (Blue/Orange = H_a, Green = H_c, Red = Me_b)

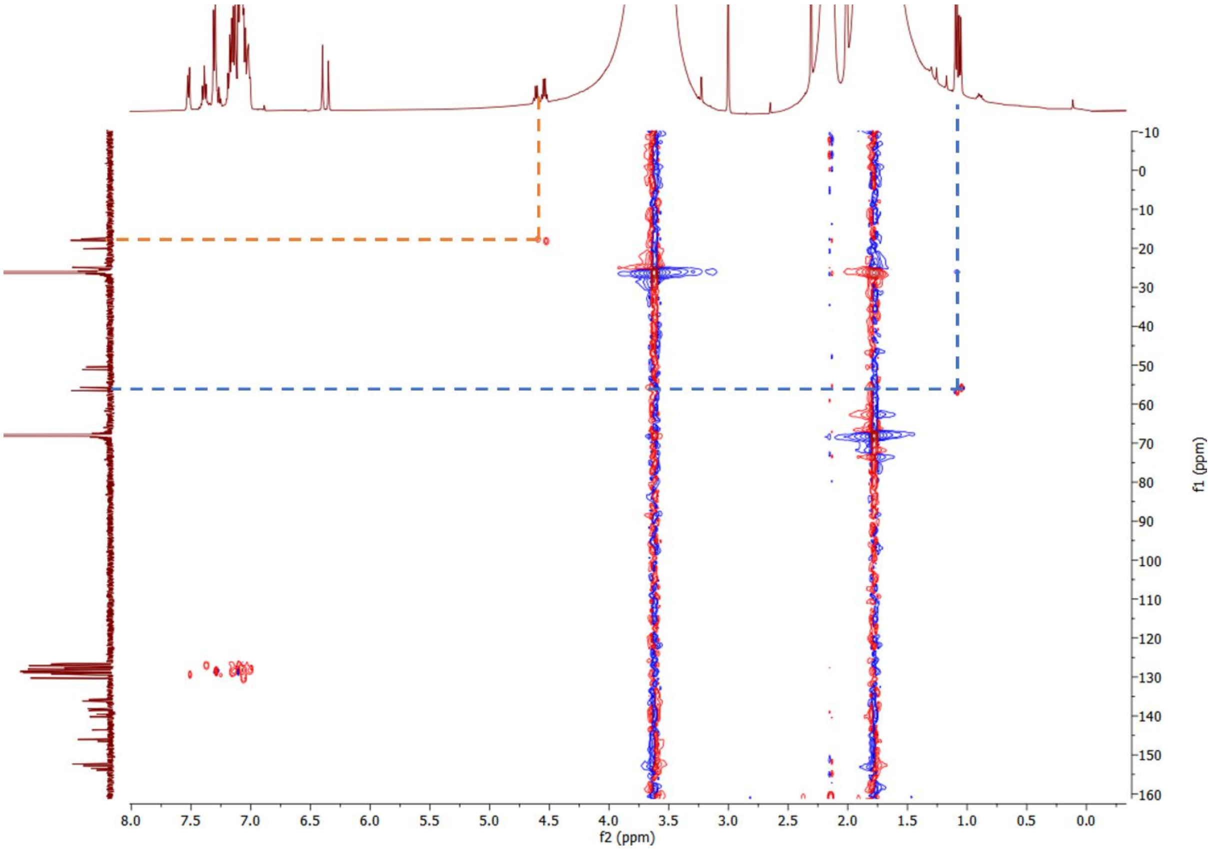

Figure S36: 500 MHz ^{1}H-^{13}C H2BC spectrum of *in-situ* formed 1,5-Me_2-1,3,4,6-Ph_4PnH in THF-H_8 (Orange = H_c, Blue = Me_b)

1,5-di(trimethyl)silyl-1,3,4,6-tetraphenyl-5-hydropentalene

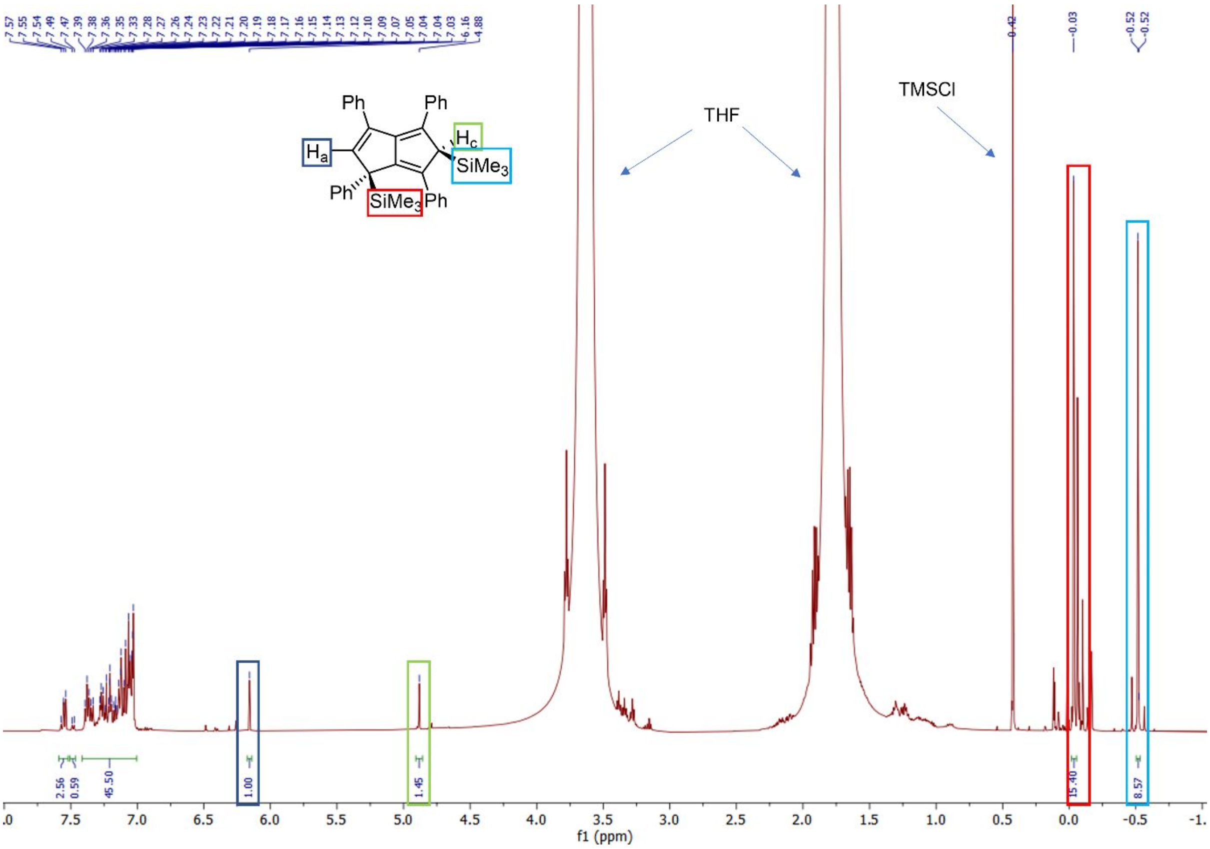

Figure S37: 500 MHz ^{1}H NMR spectrum of *in*-situ formed 1,5-(TMS)$_2$-1,3,4,6-Ph$_4$PnH in THF-H$_8$

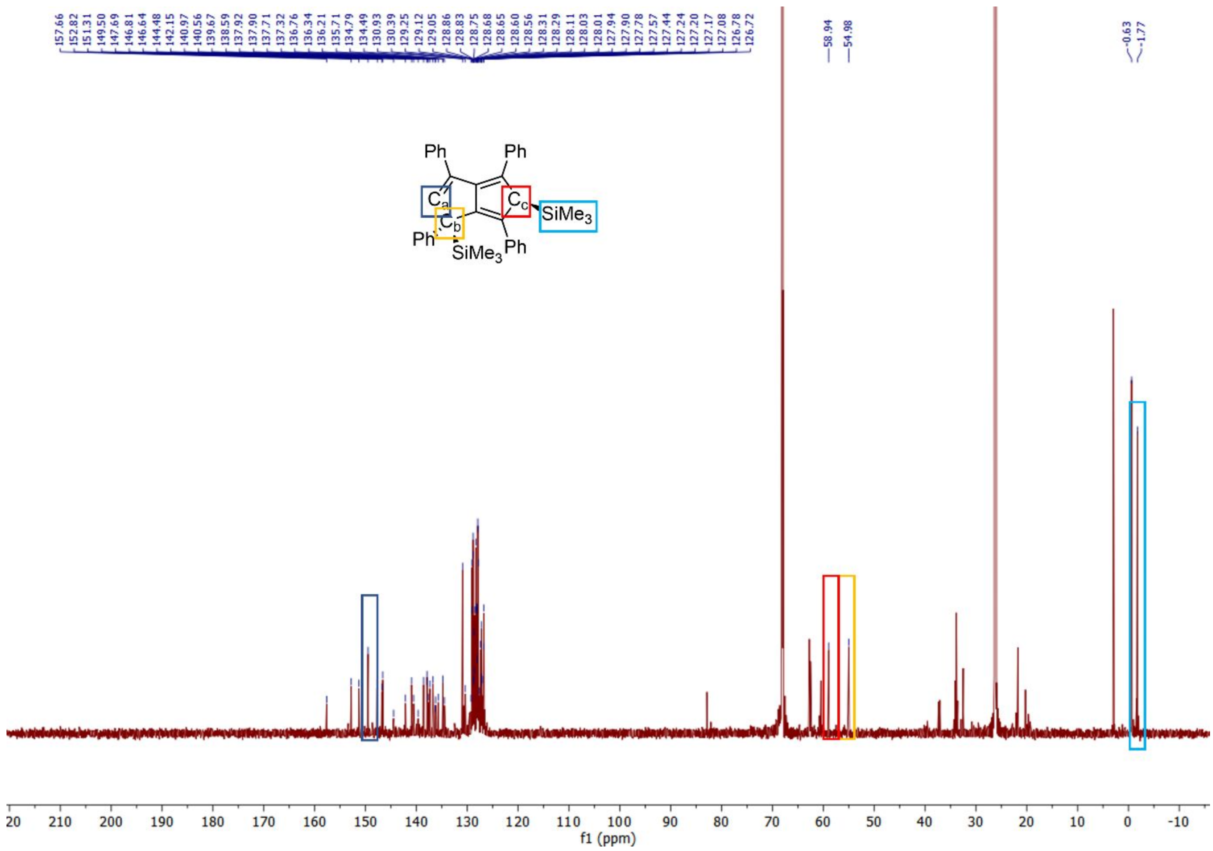

Figure S38: 126 MHz $^{13}C\{^{1}H\}$ NMR spectrum of *in*-situ formed 1,5-(TMS)$_2$-1,3,4,6-Ph$_4$PnH in THF-H$_8$

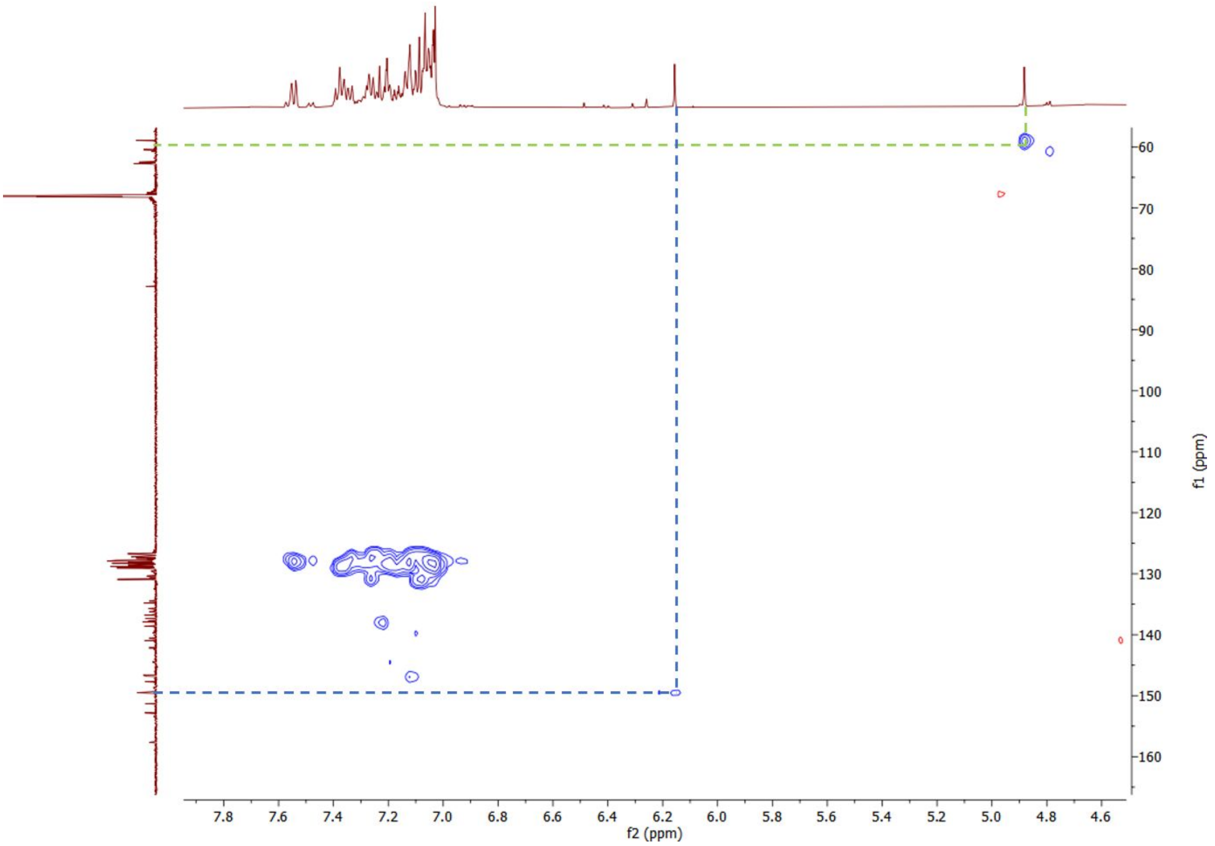

Figure S39: 500 MHz ^{1}H-^{13}C HSQC spectrum of *in*-situ formed 1,5-(TMS)$_2$-1,3,4,6-Ph$_4$PnH in THF-H$_8$ (Blue = H$_a$, Green = H$_c$)

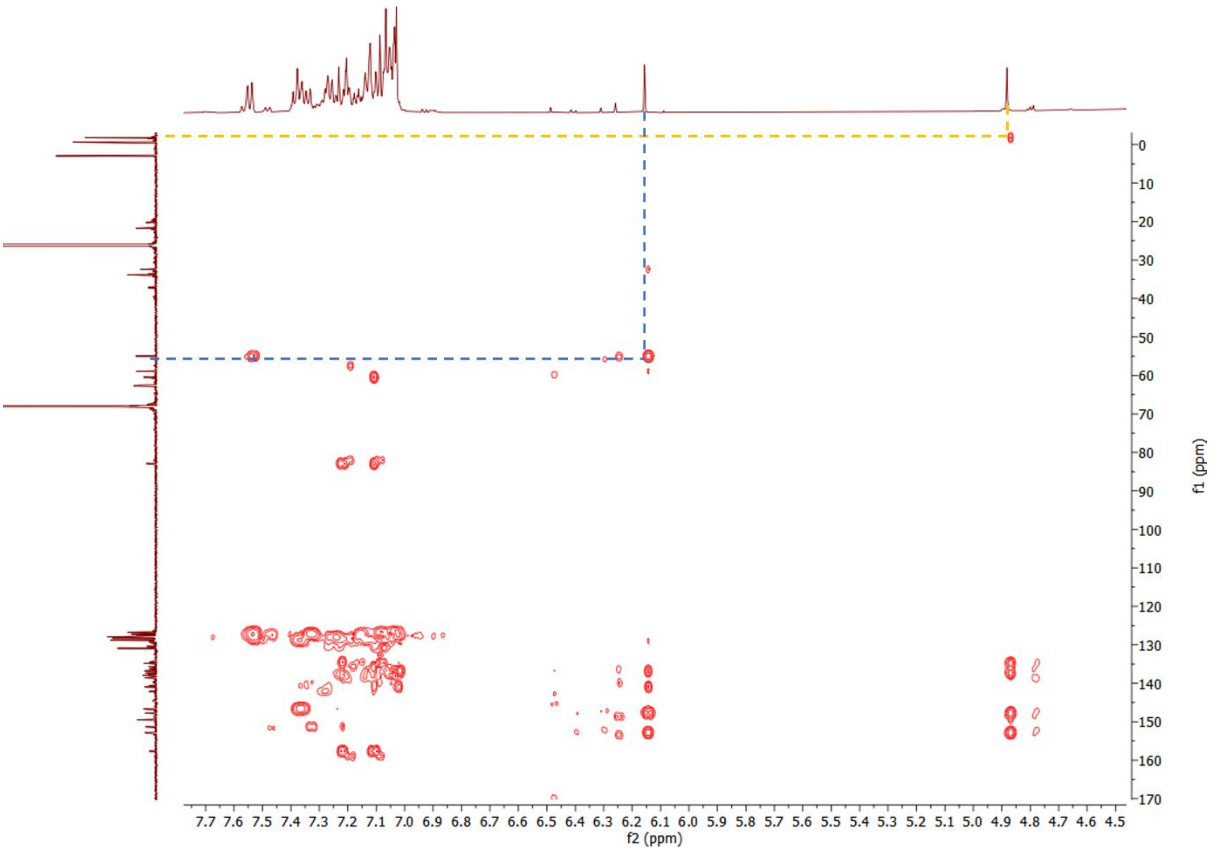

Figure S40: 500 MHz ^1H-^{13}C HMBC NMR spectrum of *in*-situ formed 1,5-$(TMS)_2$-1,3,4,6-Ph_4PnH in THF-H_8 (Blue = H_a, Green = H_c)

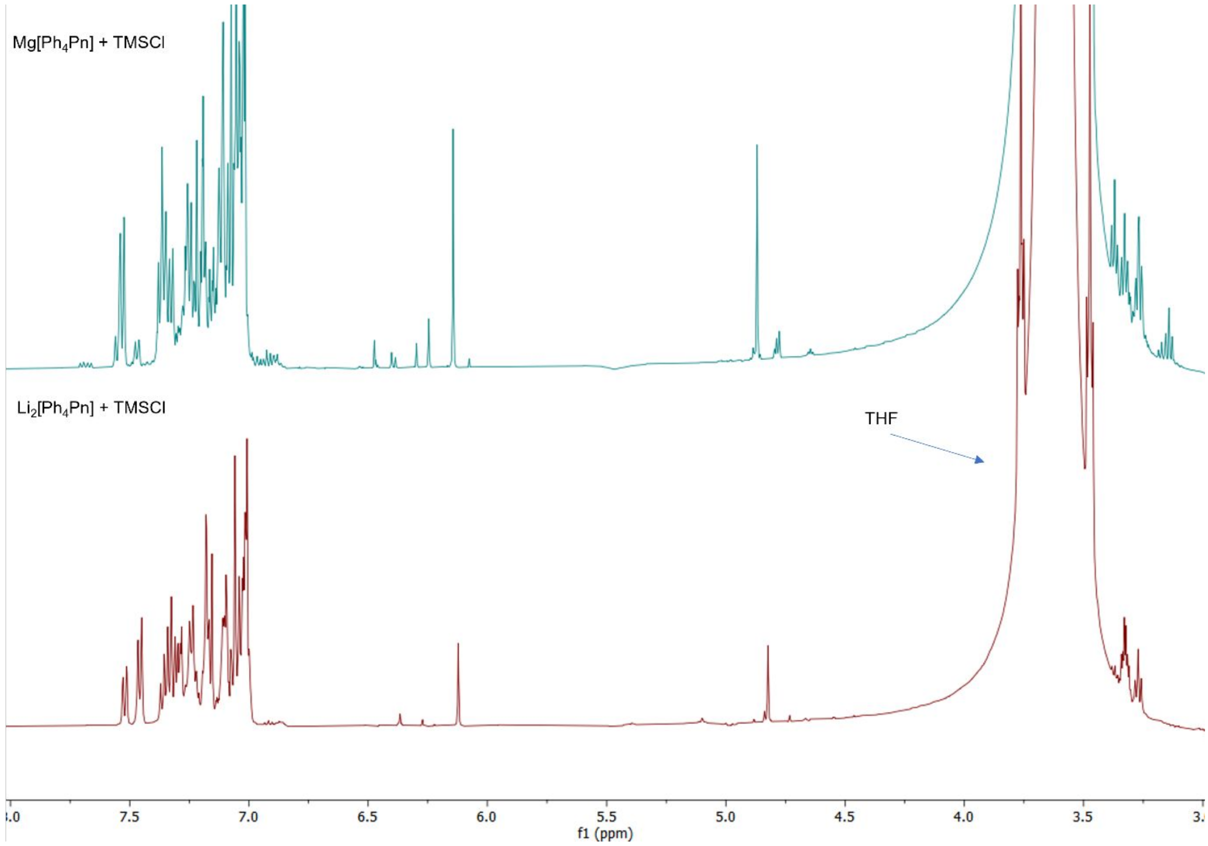

Figure S41: 500 MHz ^1H NMR spectra of the reaction between $[Mg(THF)_3][Ph_4Pn]$ and TMSCl (top) and $Li_2[Ph_4Pn]$ and TMSCl (bottom) in THF-H_8

Crystallographic Data

Magnesium 1,3,4,6-tetraphenylhydropentalenide $[Mg_2Cl_3(THF)_6][Ph_4PnH]$ (1)

**CCDC**	**2270784**	
Identification code	s22uh18	
Empirical formula	$C_{56} H_{71} Cl_3 Mg_2 O_6$	
Formula weight	995.09	
Temperature	150.01(10) K	
Wavelength	1.54184 Å	
Crystal system	Monoclinic	
Space group	I2/a	
Unit cell dimensions	a = 20.7950(2) Å	α = 90°
	b = 14.82467(13) Å	β = 100.5398(11)°
	c = 34.6702(4) Å	γ = 90°
Volume	10507.76(19) $Å^3$	
Z	8	
Density (calculated)	1.258 Mg/m^3	
Absorption coefficient	2.196 mm^{-1}	
F(000)	4240	
Crystal size	0.400 x 0.040 x 0.030 mm^3	
Theta range for data collection	3.683 to 72.943°	
Index ranges	-25<=h<=25, -11<=k<=18, -42<=l<=42	
Reflections collected	57836	
Independent reflections	10416 [R(int) = 0.0384]	
Completeness to theta = 67.684°	100.0 %	
Absorption correction	Semi-empirical from equivalents	
Max. and min. transmission	1.00000 and 0.79390	
Refinement method	Full-matrix least-squares on F^2	
Data / restraints / parameters	10416 / 96 / 680	
Goodness-of-fit on F^2	1.012	
Final R indices [I>2sigma(I)]	R1 = 0.0397, wR2 = 0.0955	
R indices (all data)	R1 = 0.0550, wR2 = 0.1035	
Extinction coefficient	n/a	
Largest diff. peak and hole	0.268 and -0.249 e.$Å^{-3}$	

Magnesium 1,3,4,6-tetraphenylpentalenide [Mg(THF)$_3$][Ph4Pn] (2)

**CCDC**	**2270785**	
Identification code	s21uh1	
Empirical formula	$C_{44} H_{46} Mg O_3$	
Formula weight	647.12	
Temperature	150.00(10) K	
Wavelength	1.54184 Å	
Crystal system	Monoclinic	
Space group	Ia	
Unit cell dimensions	a = 16.24340(10) Å	α = 90°.
	b = 14.72330(10) Å	β = 118.7980(10)°.
	c = 16.57760(10) Å	γ = 90°.
Volume	3474.31(5) $Å^3$	
Z	4	
Density (calculated)	1.237 Mg/m^3	
Absorption coefficient	0.749 mm^{-1}	
F(000)	1384	
Crystal size	0.506 x 0.453 x 0.374 mm^3	
Theta range for data collection	4.275 to 72.871°.	
Index ranges	-20<=h<=20, -18<=k<=18, -20<=l<=19	
Reflections collected	34854	
Independent reflections	6747 [R(int) = 0.0207]	
Completeness to theta = 67.684°	100.0 %	
Absorption correction	Semi-empirical from equivalents	
Max. and min. transmission	1.00000 and 0.30112	
Refinement method	Full-matrix least-squares on F^2	
Data / restraints / parameters	6747 / 2 / 433	
Goodness-of-fit on F^2	1.037	
Final R indices [I>2sigma(I)]	R1 = 0.0354, wR2 = 0.0957	
R indices (all data)	R1 = 0.0356, wR2 = 0.0959	
Absolute structure parameter	0.033(9)	
Extinction coefficient	n/a	
Largest diff. peak and hole	0.540 and -0.349 e.$Å^{-3}$	

Di(butylmagnesium) 1,3,4,6-tetraphenylpentalenide $[Mg(^{n}Bu)(THF)_2]_2[Ph_4Pn]$ (3)

**CCDC**	**2270786**	
Identification code	s22uh7	
Empirical formula	$C_{56} H_{72} Mg_2 O_4$	
Formula weight	857.75	
Temperature	150.01(10) K	
Wavelength	1.54184 Å	
Crystal system	Triclinic	
Space group	P-1	
Unit cell dimensions	a = 10.6749(3) Å	α = 81.987(2)°.
	b = 10.8372(3) Å	β = 79.022(2)°.
	c = 11.2199(3) Å	γ = 71.811(3)°.
Volume	1206.11(6) $Å^3$	
Z	1	
Density (calculated)	1.181 Mg/m^3	
Absorption coefficient	0.789 mm^{-1}	
F(000)	464	
Crystal size	0.180 x 0.100 x 0.100 mm^3	
Theta range for data collection	4.028 to 72.979°.	
Index ranges	-13<=h<=13, -12<=k<=13, -13<=l<=13	
Reflections collected	25550	
Independent reflections	4805 [R(int) = 0.0293]	
Completeness to theta = 67.684°	100.0 %	
Absorption correction	Semi-empirical from equivalents	
Max. and min. transmission	1.00000 and 0.30498	
Refinement method	Full-matrix least-squares on F^2	
Data / restraints / parameters	4805 / 0 / 288	
Goodness-of-fit on F^2	1.047	
Final R indices [I>2sigma(I)]	R1 = 0.0363, wR2 = 0.0934	
R indices (all data)	R1 = 0.0384, wR2 = 0.0951	
Extinction coefficient	n/a	
Largest diff. peak and hole	0.228 and -0.220 e.$Å^{-3}$	

Magnesium 1,3,4,6-tetraphenylpentalenide from interconversion

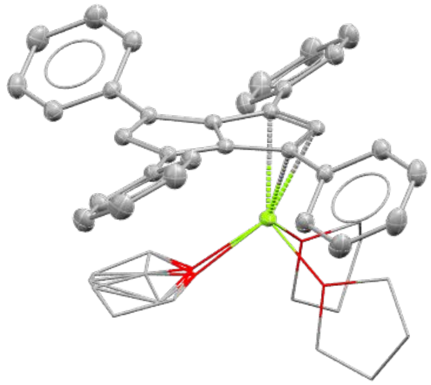

Figure 42: X-ray crystal structure of [Mg(THF)$_3$][Ph4Pn]$_*$(THF) formed from the interconversion of [Mg-nBu(THF)$_2$]$_2$[Ph$_4$Pn]. Thermal ellipsoids shown at the 30% probability level. Hydrogen atoms and solvent molecule of THF are omitted for clarity.

**CCDC**	**2270787**	
Identification code	s22uh28	
Empirical formula	$C_{52} H_{62} Mg O_5$	
Formula weight	791.32	
Temperature	150.00(10) K	
Wavelength	1.54184 Å	
Crystal system	Orthorhombic	
Space group	$Cmc2_1$	
Unit cell dimensions	a = 27.52634(19) Å	a= 90°.
	b = 10.49333(8) Å	b= 90°.
	c = 15.09248(10) Å	g = 90°.
Volume	4359.36(5) Å^3	
Z	4	
Density (calculated)	1.206 Mg/m^3	
Absorption coefficient	0.720 mm^{-1}	
F(000)	1704	
Crystal size	0.300 x 0.200 x 0.180 mm^3	
Theta range for data collection	4.510 to 72.907°.	
Index ranges	-34<=h<=34, -12<=k<=10, -18<=l<=18	
Reflections collected	38395	
Independent reflections	4417 [R(int) = 0.0244]	
Completeness to theta = 67.684°	100.0 %	
Absorption correction	Semi-empirical from equivalents	
Max. and min. transmission	1.00000 and 0.89543	
Refinement method	Full-matrix least-squares on F^2	

Data / restraints / parameters	4417 / 224 / 382
Goodness-of-fit on F^2	1.042
Final R indices [I>2sigma(I)]	R1 = 0.0362, wR2 = 0.0976
R indices (all data)	R1 = 0.0367, wR2 = 0.0982
Absolute structure parameter	0.28(6)
Extinction coefficient	n/a
Largest diff. peak and hole	0.295 and -0.226 e.Å^{-3}
